# Recellularization of rat liver: An in vitro model for assessing human drug metabolism and liver biology

**DOI:** 10.1371/journal.pone.0191892

**Published:** 2018-01-29

**Authors:** Matthew J. Robertson, Benjamin Soibam, Jacqueline G. O’Leary, Luiz C. Sampaio, Doris A. Taylor

**Affiliations:** 1 Scientific Stem Cell, Texas Heart Institute, Houston, Texas, United States of America; 2 Department of Computer Science and Engineering Technology, University of Houston-Downtown, Houston, Texas, United States of America; 3 Annette C. & Harold C. Simmons Transplant Institute, Baylor University Medical Center, Dallas, Texas, United States of America; 4 Regenerative Medicine Research Laboratories, Texas Heart Institute, Houston, Texas, United States of America; Michigan Technological University, UNITED STATES

## Abstract

Liver-like organoids that recapitulate the complex functions of the whole liver by combining cells, scaffolds, and mechanical or chemical cues are becoming important models for studying liver biology and drug metabolism. The advantages of growing cells in three-dimensional constructs include enhanced cell-cell and cell-extracellular matrix interactions and preserved cellular phenotype including, prevention of de-differentiation. In the current study, biomimetic liver constructs were made via perfusion decellularization of rat liver, with the goal of maintaining the native composition and structure of the extracellular matrix. We optimized our decellularization process to produce liver scaffolds in which immunogenic residual DNA was removed but glycosaminoglycans were maintained. When the constructs were recellularized with rat or human liver cells, the cells remained viable, capable of proliferation, and functional for 28 days. Specifically, the cells continued to express cytochrome P450 genes and maintained their ability to metabolize a model drug, midazolam. Microarray analysis showed an upregulation of genes involved in liver regeneration and fibrosis. In conclusion, these liver constructs have the potential to be used as test beds for studying liver biology and drug metabolism.

## Introduction

A goal of liver tissue engineering is to generate artificial, de novo functional liver tissue. A quickly growing area of research in this field is the development of *in vitro* models to advance our knowledge of liver biology [1, 2] and expedite drug development [3, 4]. These in vitro models have already increased the efficiency of drug screening, thereby accelerating preclinical studies in the drug development process. In addition, in vitro models have the potential to dramatically reduce the cost of bringing drugs to market [5]. Despite these recent advances, there is still a need for a tissue-engineered liver model that recapitulates the *in vivo* microenvironment. Such a model would likely be more predictive than those that are currently the standard in regulatory studies.

Hepatocytes are the most prevalent parenchymal cell type, comprising 60 to 80% of the liver mass. These cells perform most of the non-immunologic functions of the liver [6]. Hepatocytes rapidly lose their phenotype in cell culture [7]. However, preserving the microenvironment of the liver (e.g., three-dimensional structure, cell-cell and cell-extracellular matrix [ECM] interactions, chemical cues, and mechanical properties) helps hepatocytes retain their phenotype in cell culture [5][8–10]. For example, it is well established that restoring cell-ECM interactions by culturing hepatocytes in the presence of purified ECM proteins, such as collagen, or complex protein mixtures, such as Matrigel or liver ECM gels improves hepatocyte function and helps the cells maintain their phenotype. Furthermore, hepatocytes are better able to maintain their differentiated phenotype when grown in three-dimensional cultures rather than in two-dimensional cultures [8, 9]. Conversely, culturing hepatocytes on a stiff substrate leads to their de-differentiation and proliferation, suggesting that the mechanical environment found in the liver is also important when culturing these cells [5]. Therefore, the ideal matrix for liver tissue engineering should have the same three-dimensional architecture, chemical composition, and mechanical properties as native liver so that hepatocytes maintain their mature phenotype (e.g., hepatocyte zonation within the liver, cell polarization, and function) when cultured on it. Perfusion-decellularized liver scaffolds [11] are ideally suited to meet these requirements.

Decellularized livers not only provide three-dimensional scaffolds that are similar to the native liver, but they also contain the ECM proteins found in the native liver, including collagen, fibronectin, and basement membrane proteins [12–22]. Furthermore, decellularized liver scaffolds retain some of the glycosaminoglycans (GAGs), cytokines, and growth factors present in the native liver, including fibroblast growth factor-basic (bFGF) [16], hepatocyte growth factor (HGF) [16], and vascular endothelial growth factor (VEGF) [14]. These scaffolds, by virtue of being generated from the native liver, have an acellular vascular tree with patent vessel conduits [12, 23]. This acellular vasculature is beneficial for introducing cells into the decellularized liver and for perfusing cell culture medium throughout the tissue to maintain the viability and phenotype of the cells under physiologic pulsatile fluid flow. Proper perfusion of medium across the cells creates the potential for establishing the nutrient and oxygen gradients found in the native liver microenvironment, which is important for maintaining zonation within the liver. Establishing proper fluid shear stress on the liver cells is also important because metabolic function, cell viability, and adhesion molecule expression are altered under high shear stress conditions [8, 24].

Because the liver must have the appropriate three-dimensional structure, mechanical properties, and chemical composition to perform its basic functions, we sought to produce a liver construct that maintains these properties and the native cues necessary for proper cell function in a culture system that mimics the *in vivo* microenvironment. Our findings suggest that livers that have been perfusion-decellularized and recellularized can be used as a model system for studying changes in cell phenotype in the context of drug metabolism and liver diseases.

## Materials and methods

### Animal use

All experiments were performed in accordance with the US Animal Welfare Act and were approved by the Institutional Animal Care and Use Committee at the University of Minnesota. Sprague Dawley rats (Harlan Laboratories), ranging in age from 13 to 28 weeks old, were anesthetized with ketamine (100 mg/kg) (Phoenix Pharmaceutical) and xylazine (10 mg/kg) (Phoenix Pharmaceutical) before any procedures were performed. They were then systemically heparinized via femoral artery or infragastric vein injection, and their livers were subsequently removed.

### Decellularization

The caudate lobes of the rat livers were isolated by placing 7–0 proline sutures loosely around the branches of the portal vein leading to the other lobes. Next, the portal vein was cannulated, and each liver was perfused with phosphate-buffered saline (PBS) and placed in a bioreactor. Three different decellularization procedures were evaluated: (1) perfusion with 3.2 L of 1% sodium dodecyl sulfate (SDS) in water, followed by 60 mL of deionized water, then 60 mL of 1% Triton X-100 (Sigma), and finally 60 mL of deionized water; (2) perfusion with 6.4 L of 1% SDS in water, 60 mL of deionized water, 60 mL of 1% Triton X-100 (Sigma), and then 60 mL of deionized water; and (3) perfusion with 3.2 L of 1% SDS in water, followed by 120 mL of water, and then recirculation of a DNase (Roche) (200 units/mL) through the scaffolds for 4 hours at room temperature. All perfusions were started at an initial pressure of 20 mmHg and were gravity driven. After the decellularization procedures, the scaffolds were washed twice by recirculating 500 mL of PBS containing penicillin/streptomycin (Life Technologies) for at least 6 hours per wash. Next, the 7–0 sutures that were placed around the portal vein branches leading to all the lobes except the caudate lobes were tied. Each acellular scaffold was then placed into a tissue culture incubator (37° C), attached to a peristaltic pump, and perfused with cell culture medium at a rate of 1 mL/min for 24 hours before recellularization.

### Residual DNA quantification

Samples (decellularized scaffolds or whole liver biopsies) were hydrolyzed with 1N NaOH, and the DNA content was then analyzed by using Hoechst 33258 (Life Technologies). For this analysis, the diluted hydrolyzed samples were mixed with 2X Hoechst dye reagent (200 ng/mL Hoechst 33258 in TNE buffer), the fluorescence intensity of the samples was measured by using a fluorometer, and the DNA content was quantified using a standard curve produced with calf thymus DNA.

### Residual GAG quantification

Samples (decellularized scaffolds or whole liver biopsies) were hydrolyzed with 1N NaOH. GAG content was analyzed by using the Biocolor Blyscan Assay Kit (Accurate Chemical). Briefly, samples, blanks, and standards were incubated in Biocolor Assay Dye Reagent for 2 hours at room temperature. The samples were then centrifuged, and the supernatants were decanted. Pellets were suspended in the Biocolor Blyscan GAG Assay Dye Dissociative Reagent, and GAG content was measured using a spectrophotometer.

### Human liver cells

A crude, prefiltered preparation of human cells containing hepatocytes and nonparenchymal cells was obtained from CellzDirect, centrifuged, and the shipping buffer was removed. Cells were suspended gently in cell culture medium. Cell concentration and viability were quantified using a hemocytometer and 0.4% Trypan Blue (Life Technologies). In all human liver cell recellularization experiments, twenty million cells were used to recellularize the liver constructs.

### Rat liver cells

Liver cells were isolated from anesthetized and heparinized Sprague Dawley rats by using a two-step collagenase perfusion protocol. Briefly, the portal vein was surgically exposed and catheterized and then perfused with a HEPES-buffered solution containing 142 mM NaCl, 6.7 mM KCl, 5 mM EGTA, and 10 mM HEPES (pH = 7.6). The perfusate was then changed to a HEPES-buffered collagenase solution containing 100 U/mL collagenase (Worthington), 67 mM NaCl, 6.7 mM KCl, 5 mM CaCl_2_, and 100 mM HEPES (pH = 7.6). After collagenase-mediated liver digestion and disruption of Glisson’s capsule, the cells were filtered through a 100-micron filter and then washed with EGTA-containing buffer. Viable cells were isolated by mixing the cell suspension with Percoll (Sigma) to form a 40% isotonic Percoll solution (Sigma). This solution was then centrifuged at 70 g to remove the non-viable cells [25]. The viable cells were then washed and diluted in preparation for recellularization of the acellular liver scaffolds.

### Recellularization

In our experiments, we recellularized scaffolds with either rat liver cells (one million or twenty million cells) or human liver cells (twenty million cells). For experiments in which the constructs were recellularized with twenty million cells, the liver cells were diluted with culture medium to four million cells per mL. For experiments in which constructs were recellularized with only one million cells, the cells were diluted to two hundred thousand cells per mL. Scaffolds were first removed from their bioreactors and perfused with PBS. Using a portal vein catheter, 5 mL of cell stock (i.e., either one million or twenty million cells) was perfused into the scaffold. Afterwards, the recellularized scaffolds were placed back into the bioreactors and cultured for the indicated duration.

### Liver bioreactors

Recellularized liver constructs were cultured in a bioreactor that consisted of a sterilized Erlenmeyer flask with a port at the bottom. The flask was corked with a sterile rubber stopper to ensure sterility. An 18-gauge needle was pushed through the rubber stopper, and the cannulated liver portal veins were attached to the needle. In all experiments, liver constructs were perfused at a rate of 1 mL/min by using a Masterflex C/L pump (Cole-Parmer) that was attached to the 18-gauge needle. The medium was oxygenated by bubbling with humidified carbogen. The entire liver bioreactor (medium reservoir, flask containing the liver construct, carbogen humidification pump, and pump) was kept in a tissue culture incubator. Before the bioreactors were set up, all tubing, attachments, flasks, and stoppers were sterilized by autoclaving.

### Culture media

Livers recellularized with rat cells were perfused with Williams’ Medium E (Sigma) containing 2 mM GlutaMax-1 (Life Technologies), 0.1 mM MEM non-essential amino acids solution (Sigma), 10% fetal bovine serum (Hyclone), 10 μg/mL insulin (Sigma), 1X penicillin/streptomycin (Life Technologies), and 1 μM dexamethasone (Sigma).

Scaffolds seeded with human liver cells were perfused with Williams’ Medium E (Sigma) containing 2 mM GlutaMax-1 (Life Technologies), 0.1 mM MEM nonessential amino acids solution (Sigma), 1X ITS+1 (Sigma), 2.0 ng/mL HGF (Peprotech), 2.0 ng/mL epidermal growth factor (EGF) (Peprotech), 1X penicillin/streptomycin (Life Technologies), and 0.1 μM dexamethasone (Sigma).

### Human albumin measurements

A direct enzyme-linked immunosorbent assay (ELISA) was used to determine the concentration of albumin in the culture medium. A human serum albumin antibody conjugated to horseradish peroxidase was used per the manufacturer’s guidelines (Bethyl Laboratories, Inc.). Albumin production was normalized by the number of cells seeded onto the scaffold.

### Human urea measurements

The urea level in medium samples collected from livers recellularized with human liver cells was quantified at the indicated time points by using a Blood Urea Nitrogen Kit per the manufacturer’s guidelines (Pointe-Scientific, B7551-120). Urea measurements were normalized by the number of cells seeded onto the scaffold.

### Glucose 6-phosphate dehydrogenase (G6PDH) activity

A Vybrant Cytotoxicity Assay Kit (Life Technologies) was used per the manufacturer’s guidelines to measure G6PDH activity in medium harvested from constructs recellularized with human liver cells at the indicated time points.

### Clinical chemistry

For all constructs recellularized with rat liver cells, the concentration of urea, albumin, glucose and the aspartate transaminase (AST) activity level in medium samples were measured by the University of Minnesota’s Veterinary Diagnostic Lab (St. Paul, MN). All clinical chemistry measurements were normalized by the number of cells seeded onto the scaffold.

### Immunostaining

Samples from scaffolds recellularized with human or rat liver cells were fixed in formalin and embedded in paraffin. The samples were then sectioned, dewaxed, and hydrated in a graded ethanol series. Before the sections were immunostained, antigen retrieval was performed, which included boiling the samples in a Tris EDTA buffer (10 mM Tris base, 1 mM EDTA, and 0.05% Tween-20 [pH = 9.0]). The following primary antibodies were used for immunostaining: rabbit polyclonal IgG anti-proliferating cell nuclear antigen (PCNA) (Santa Cruz), rabbit polyclonal anti-collagen I (Abcam), and rabbit polyclonal anti-elastin (Abcam).

When immunostaining for PCNA, samples were washed twice in Tris-buffered saline (TBS) (pH = 8.4) with 0.025% Tween-20 and blocked in Tris-buffered saline with 1% bovine serum album. The samples were incubated with primary antibody in blocking buffer overnight at 4°C. The next day, the samples were washed twice in TBS containing Tween-20, incubated with secondary antibody (DyLight 594 donkey anti-rabbit IgG, Jackson ImmunoResearch) in TBS, washed 3 times in TBS, and mounted using Vectashield HardSet Mounting Medium with DAPI (Vector Laboratories).

A similar procedure was used for type I collagen and elastin staining, but the following modifications were made: slides were washed with PBS containing 0.05% Tween-20, blocked with PBS containing 5% normal donkey serum, incubated with the primary antibodies in PBS containing 5% donkey serum, and incubated with the secondary antibody Texas Red dye–conjugated AffiniPure donkey anti-rabbit IgG (Jackson ImmunoResearch).

For the immunohistochemistry analyses, antigen retrieval was performed by boiling the samples in 10 mM citrate buffer (pH = 6.0) with 0.05% Tween-20. The Universal RTU VECTASTAIN Elite ABC Kit (Vector Laboratories) was used per the manufacturer’s guidelines to stain the slides. Slides were incubated with rabbit polyclonal IgG anti-CYP450 3A4 (Abcam) or mouse monoclonal IgG anti-CYP450 1A2 (Abcam). Finally, stained sections were incubated for 10 mins with Vector Novared (Vector Laboratories), which was used as the peroxidase substrate. Slides were mounted with Vectamount (Vector Laboratories).

### PCNA quantification

At the indicated time points, at least 5 fields of view from the sections of recellularized liver constructs stained with PCNA and DAPI were randomly selected and imaged. Within these randomly selected fields of view, the number of cells stained positive for both PCNA and DAPI were counted. For each time point, at least 4 constructs were analyzed.

### Histology

For all samples (liver biopsies, decellularized scaffolds, and recellularized scaffolds), Masson’s trichrome, hematoxylin and eosin, and silver reticulin staining were performed by the University of Minnesota’s Lillehei Heart Institute Histology and Microscopy Core by using standard protocols.

### TUNEL assay

The DeadEnd Colorimetric TUNEL system (Promega) was used to stain for nicked DNA. Further details are in the Supplemental Materials and Methods. The assay was performed according to the manufacturer’s guidelines for paraffin-embedded samples but with the following modifications: after the samples were deparaffinized and rehydrated, they were microwaved in a 10 mM citrate buffer solution, incubated with DyLight 594-conjugated streptavidin (Jackson ImmunoResearch), and mounted using Vectashield containing DAPI (Vector Laboratories).

### Scanning electron microscopy

Samples (decellularized scaffolds and liver biopsies) were fixed with 2.5% (v/v) glutaraldehyde (Electron Microscopy Sciences) in a buffered solution of 0.1 M sodium cacodylate (pH = 7.3) for 2 hours at room temperature. Samples were then washed with cacodylate buffer and postfixed with 1% osmium tetroxide in a buffered solution of 0.1 M sodium cacodylate (pH = 7.3) for 1 hour at room temperature. Next, samples were washed 3 more times and then dehydrated with a graded ethanol series. They were then dried in a Tousimis 780A critical-point dryer. Samples were sputter-coated with 10 nm AuPd (60%/40% alloy) by using a Denton DV-502A high-vacuum system (Denton Vacuum) and were visualized with a Hitachi S4700 scanning electron microscope.

### Drug metabolism measurements

Drug metabolism was tested in rat liver constructs recellularized with twenty million human liver cells. On days 2, 8, 14, 20, and 28 days post-recellularization, 10 mL of culture medium containing 10 μM of midazolam was recirculated through the constructs at 1 mL/min for 6 hours. Samples were then removed from the outlet of the bioreactor at the indicated time points (0 mins, 15 mins, 30 mins, 1 hr, 2 hrs, and 3 hrs), frozen, and stored at -80°C. Samples were shipped on dry ice and analyzed by CellzDirect to determine midazolam and hydroxymidazolam concentrations using mass spectrometry. Midazolam and hydroxymidazolam concentrations were normalized by the number of cells seeded onto the scaffolds.

### RNA isolation

At days 2, 15, and 28 after recellularization, liver constructs recellularized with rat liver cells were removed from their bioreactors, and biopsies were collected. In addition, we collected samples from day 0 cadaveric rat livers and freshly isolated rat liver cells for comparison. Biopsies were stored in RNAlater (Qiagen) until further analysis. RNA was isolated by using the Qiagen RNeasy Mini Kit per the manufacturer’s guidelines.

### Microarray analysis

Purified RNA was labeled and hybridized against RatRef-12 Expression BeadChips (Illumina). Processing and hybridization were performed by the University of Minnesota’s BioMedical Genomics Center. Using limma, we processed the raw data for background correction, quantile normalization, and log2 transformation [26]. For each gene, one-way analysis of variance (ANOVA) was used to test for a statistical difference in expression level between any 2 time points. Genes that showed no statistical difference between any 2 time points (ANOVA *P* value >0.05) were removed. A robust Z-score was computed for each gene across the groups. HOPACH clustering was applied to the robust Z-scores to obtain gene clusters by using cosine-similarity as the distance metric between the genes for days 2, 15, and 28 [27]. Gene ontology analysis was performed for each identified cluster of genes by using DAVID [28], with the mouse genome as the background set of genes. For the cytochrome P450 analysis, fold changes were calculated and normalized to cadaveric livers. The data is archived in the GEO database under the accession number GSE107274.

## Results

### Generation of an acellular rat liver scaffold by using perfusion decellularization

The effectiveness of perfusion decellularization depends on the chemistry of the perfusate used to remove the cells (e.g., ionic detergents, hypotonic solutions, or trypsin) and the method by which these chemicals are delivered (e.g., gravity or pump driven) [29, 30]. In this study, we evaluated 3 methods of decellularizing cadaveric rat livers: (1) 3.2 L of 1% SDS followed by 60 mL of 1% Triton X-100, (2) 6.4 L of 1% SDS followed by 60 mL of 1% Triton X-100, and (3) 3.2 L of 1% SDS followed by a DNase treatment. For all experiments, we used gravity perfusion at 20 mmHg through the portal vein.

When the livers were perfused with 3.2 L of 1% SDS followed by 60 mL of 1% Triton X-100, the cells were completely removed (S1A and S1B Fig). Scanning electron microscopy further confirmed that the cells had been removed and revealed that smooth-walled vessels had been retained within the liver parenchyma with a fibrous scaffold of ECM radiating outward from the vessels (S1C and S1D Fig). However, livers perfused with only 3.2 L of SDS retained a relatively high amount of latent immunogenic DNA; increasing the total volume of 1.0% SDS from 3.2 L to 6.4 L reduced the residual DNA content and the total amount of GAGs present in the scaffolds (Table 1). Staining for extracellular components after decellularization with 3.2 L of 1% SDS and 1% Triton X-100 showed that liver ECM components had been retained; the parenchyma stained positive for type I collagen, and the large vessels stained positive for the basement membrane protein elastin (S2A and S2B Fig). Thus, the distribution of ECM components resembled native liver, which has collagen I in the parenchyma and basement membrane protein near large vessels.

**Table 1:** Acellular matrix characterization.

Condition	% DNA content compared to cadaveric	Average mg GAG per gram wet sample weight
3.2 L of 1.0% SDS + 60 mL 1% Triton X-100 (N = 12)	12.45 ± 1.48	803.47 ± 188.7
6.4 L of 1.0% SDS + 60 mL 1% Triton X-100 (N = 8)	6.51 ± 1.00	301.5 ± 53.63
3.2 L of 1.0% SDS + DNase treatment (N = 4)	1.54 ± 0.31	2073.91 ± 190.2

Data are expressed as mean ± SEM

Although increasing the amount of SDS improved the removal of latent DNA, it had a deleterious effect on GAG content. The nonionic detergent Triton X-100 has been reported to remove GAGs [30]. Furthermore, it has been suggested that increasing the SDS treatment may increase protein denaturation and, thereby, harm any proteins retained on the scaffold or the scaffold itself [30]. Therefore, for all subsequent decellularization experiments, we used the lowest amount of SDS solution that would efficiently remove the cells (i.e., 3.2 L of 1% SDS) and did not use Triton X-100. To minimize the amount of immunogenic residual DNA, we treated the acellular scaffolds with DNase instead. Use of this protocol resulted in the lowest amount of residual DNA and the highest retention of GAGs (Table 1).

### Recellularization of the caudate lobes of acellular scaffolds with rat liver cells

Previous liver recellularization studies have focused on recellularization of the whole scaffold [12, 13, 16, 19]. It is well established that restoring cell-cell interactions improves cell function and morphology and stabilizes cellular phenotype [5, 8]. Therefore, to increase the probability of cell-cell interactions, we delivered cells to a confined space: the caudate lobes of the decellularized rat liver.

**Fig 1:**
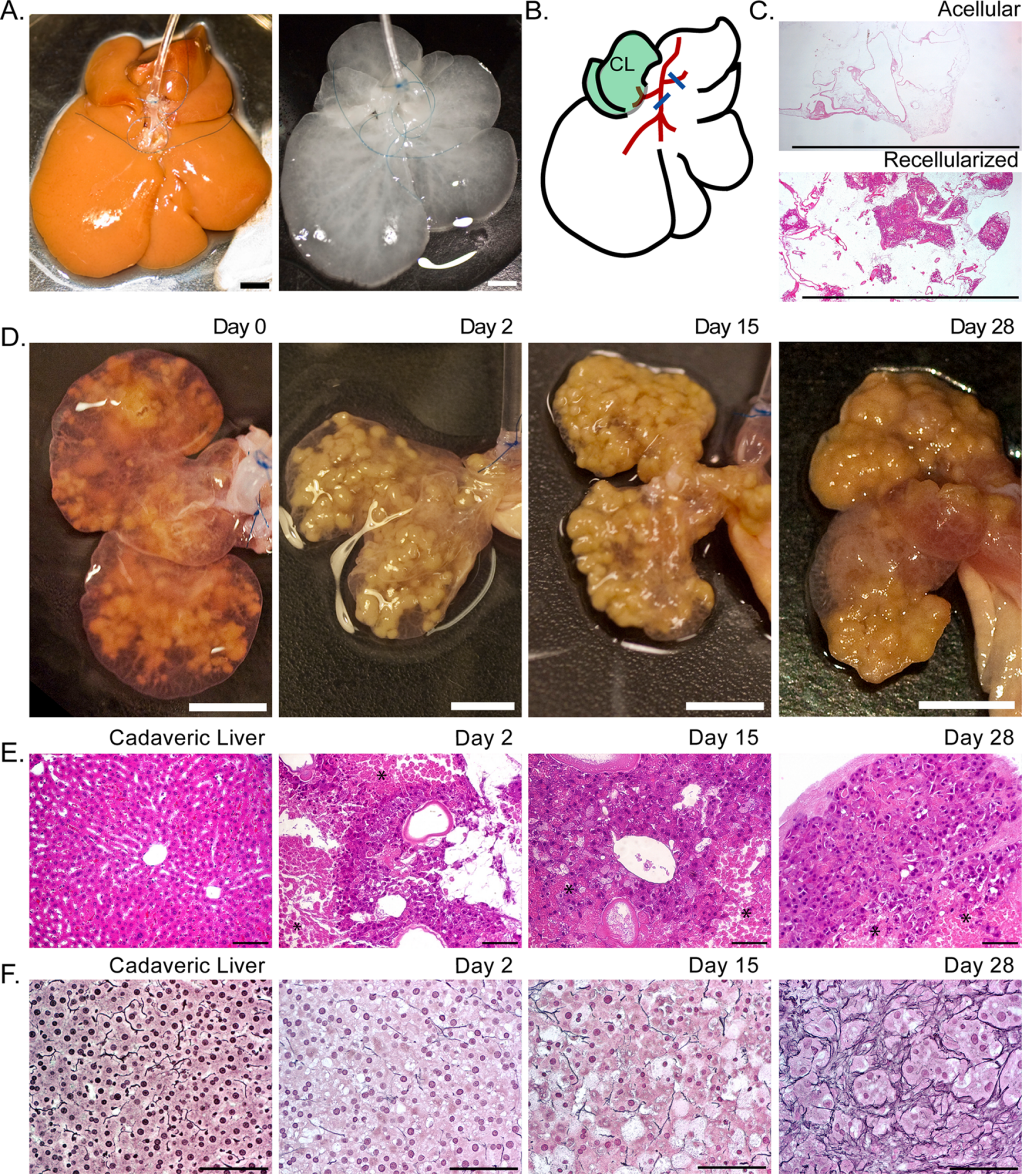
Recellularized liver lobes suitable for long-term culture were created by ligating the portal vein branches to all lobes except the caudate and perfusing twenty million rat liver cells into the acellular scaffolds. (A) Images of cadaveric rat livers before and after decellularization showing the sutures placed around the portal vein branches. (B) A schematic showing suture placement (blue lines) around the portal vein branches (red lines) for recellularization of the caudate lobes (CL, shaded green). (C) Hematoxylin and eosin staining before and 15 days after recellularization. Cell clusters can be seen in the recellularized scaffold. (D) Images of liver caudate lobes immediately after recellularization (day 0) and on days 2, 15, and 28 after recellularization. (E,F) Hematoxylin and eosin (E) and reticulin (F) staining of a cadaveric liver and recellularized liver scaffolds at days 2, 15, and 28 after recellularization. Asterisks (*) in (E) indicate regions of necrotic cells within the scaffolds. Scale bars represent 0.5 cm (A, C, and D) or 100 microns (E and F). All livers were decellularized using 3.2 L of 1% SDS followed by a DNase treatment. Images are representative of 4 liver decellularization and recellularization experiments.

Before decellularization, we placed sutures around the branches of the portal vein proximal to each liver lobe, but no suture was placed around the branch to the caudate lobes (Fig 1A and 1B). After decellularization but before recellularization, these sutures were tied, directing perfusion to the caudate lobes. To seed the scaffolds, we originally perfused twenty million rat parenchymal liver cells into the caudate lobes via the portal vein. The recellularized scaffolds were then maintained in a bioreactor within a tissue culture incubator, with culture medium perfusing the portal vein at a rate of 1 mL/min (S3A and S3B Fig). After the rat parenchymal cells were perfused into the scaffolds, they formed clusters either surrounding or near large vessels (Fig 1C). Cell retention during the initial scaffold seeding was 94.7% ± 6.0%. The average viability of the isolated hepatocytes used during the recellularization process was 91.1% ± 3.0%. After the initial perfusion of cells, the caudate lobes swelled, and overtime they condensed around the cells while the scaffold was maintained in vitro (Fig 1D). During the 28 days in culture after recellularization, the liver constructs remained intact, without any observable disruption of Glisson’s capsule surrounding the caudate liver lobes (Fig 1D). Histologic examination during the 28-day culture period showed that the engrafted cells were retained on the scaffolds (Fig 1E and 1F), but a few regions of necrotic cells could be seen distal to the large vessels (Fig 1E). Patent vessels were also observed in the recellularized scaffolds (Fig 1E). In addition, the liver constructs showed increased reticulin staining around the hepatocytes, suggestive of ECM synthesis and remodeling (Fig 1F).

**Fig 2:**
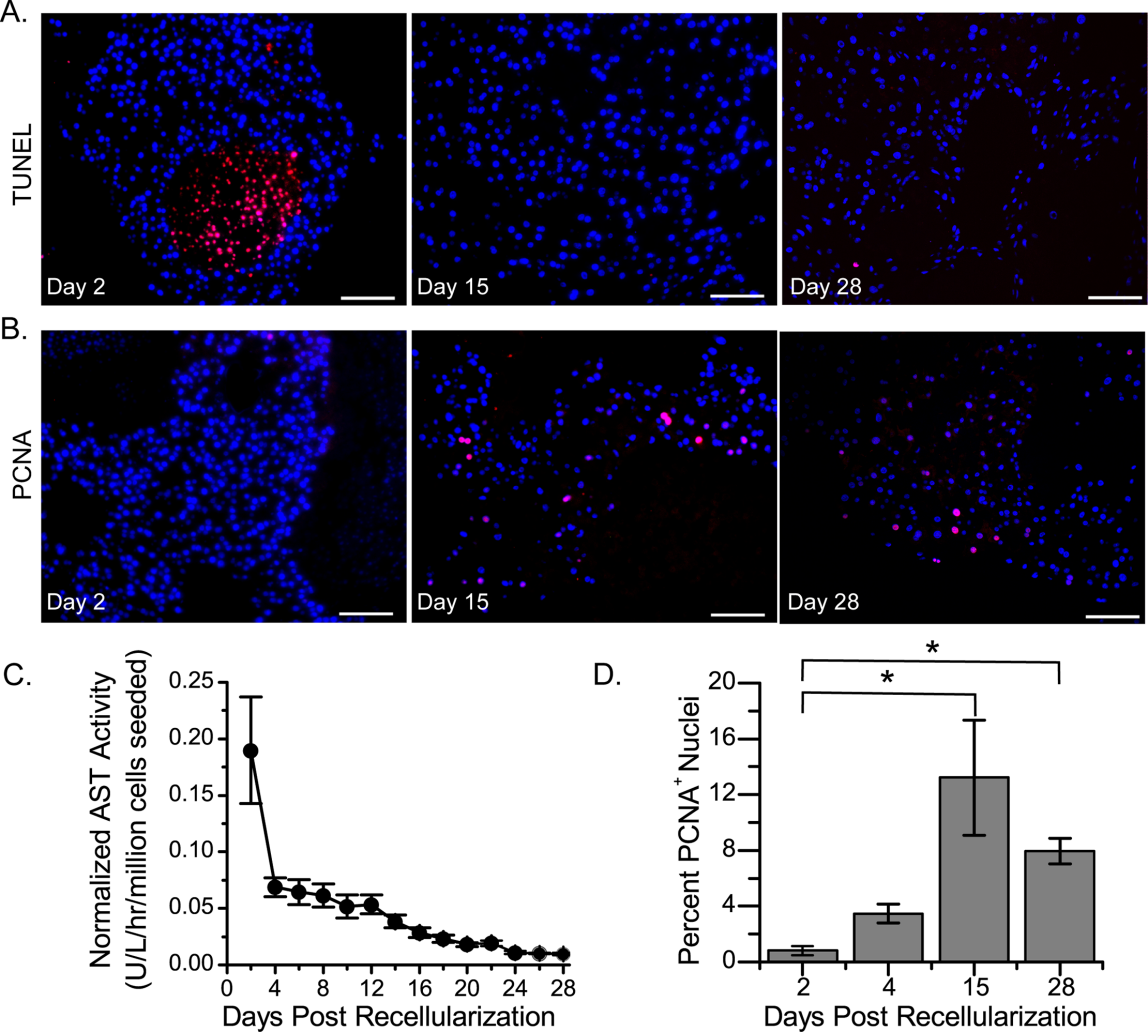
Constructs recellularized with twenty million rat liver cells and cultured for 28 days showed initial cell death followed by cell growth. (A) Representative microscopy images showing TUNEL (red) and DAPI (blue) staining in sections obtained on days 2, 15, and 28 after recellularization. (B) Representative microscopy images showing PCNA (red) and DAPI (blue) staining in sections obtained on days 2, 15, and 28 after recellularization. Scale bars in (A) and (B) represent 100 microns. (C) Graph showing aspartate transaminase (AST) activity in the cell culture medium over time. (D) Graph showing the quantification of PCNA staining. Asterisks (*) indicate a *P* value less than 0.05. The standard error of the mean is reported for 4 separate constructs (C and D). Five independent fields of view were analyzed per construct. All livers were decellularized using 3.2 L of 1% SDS followed by a DNAse treatment.

To examine the viability of the cells after construct recellularization, TUNEL staining of histologic sections was performed and AST activity in the cell culture medium measured. Two days after recellularization, many TUNEL-positive cells were present, which were typically found in the center of large cell clusters and distal from the vessels (Fig 2A). However, at days 15 and 28 after recellularization, very few TUNEL-positive cells were observed (Fig 2A). This transient increase in cell death was confirmed by measurements of AST activity in the medium, which were highest on day 2 and then rapidly decreased (Fig 2C). This decrease in cell death coincided with an increase in the expression of PCNA, a marker of cell proliferation and regeneration (Fig 2B and 2D). Compared to liver constructs that were cultured for 2 days, those that were cultured for 15 or 28 days showed a statistically significant increase in PCNA-positive cell staining. These data suggest that, after seeding, cells underwent cell death because of insufficient perfusion of cell culture medium and that some cells de-differentiated.

We then reduced the number of rat liver cells perfused into the isolated liver lobes from twenty million to one million to see if reduced cell crowding could improve cell survival by increasing cell proximity to the vessels within the liver constructs. After the perfusion of only one million cells, clusters of cells were still observed proximal to the vessels (S4A Fig), and increased reticulin staining was present between the cells (S4B Fig). Lastly, we found that cell viability, as shown by TUNEL staining at 2 days after recellularization was increased (S4C Fig).

### Functional assessment of liver constructs recellularized with rat liver cells

**Fig 3:**
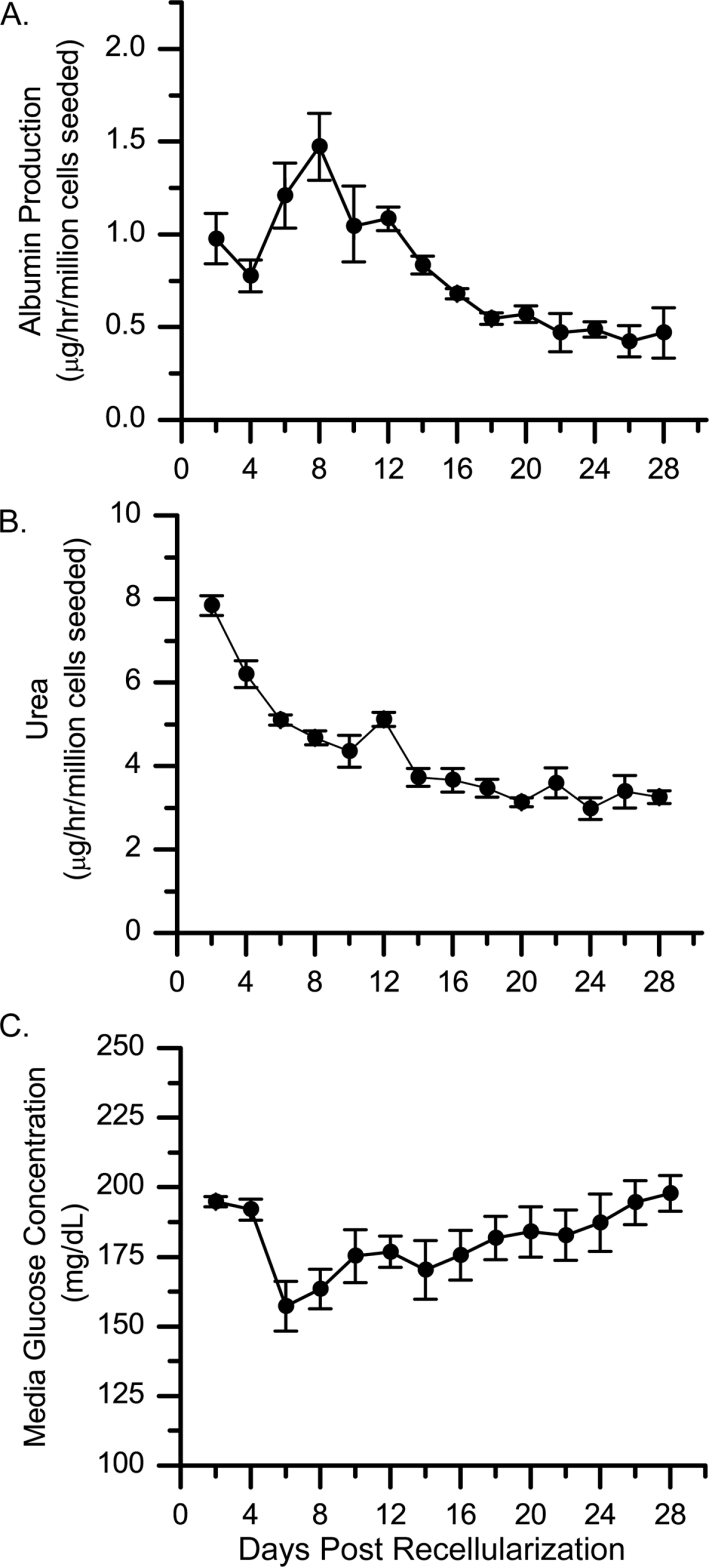
Functional activity including production of albumin and urea, and consumption of glucose in liver constructs recellularized with twenty million rat liver cells. (A-C) Albumin production (A), urea production (B), and glucose concentration (C) were measured in culture medium from recellularized liver constructs over a 28-day period. Values shown are the average for 4 different recellularized constructs. Error bars depict the standard error of the mean. These livers were decellularized using the same protocol (3.2 L of 1% SDS followed by DNase treatment).

Cell function was monitored for 28 days after liver constructs were recellularized with twenty million rat liver cells. Albumin production peaked during the first week of culture, decreased during the second week, and eventually stabilized during the last 2 weeks (Fig 3A). Conversely, urea production decreased during the first 2 weeks before stabilizing (Fig 3B). Glucose concentration in the medium decreased during the first week of culture and then returned to the initial level (Fig 3C). This transient increase in the rate of glucose consumption was suggestive of cell growth and preceded the observed increase in PCNA staining (Fig 2B and 2D).

**Fig 4:**
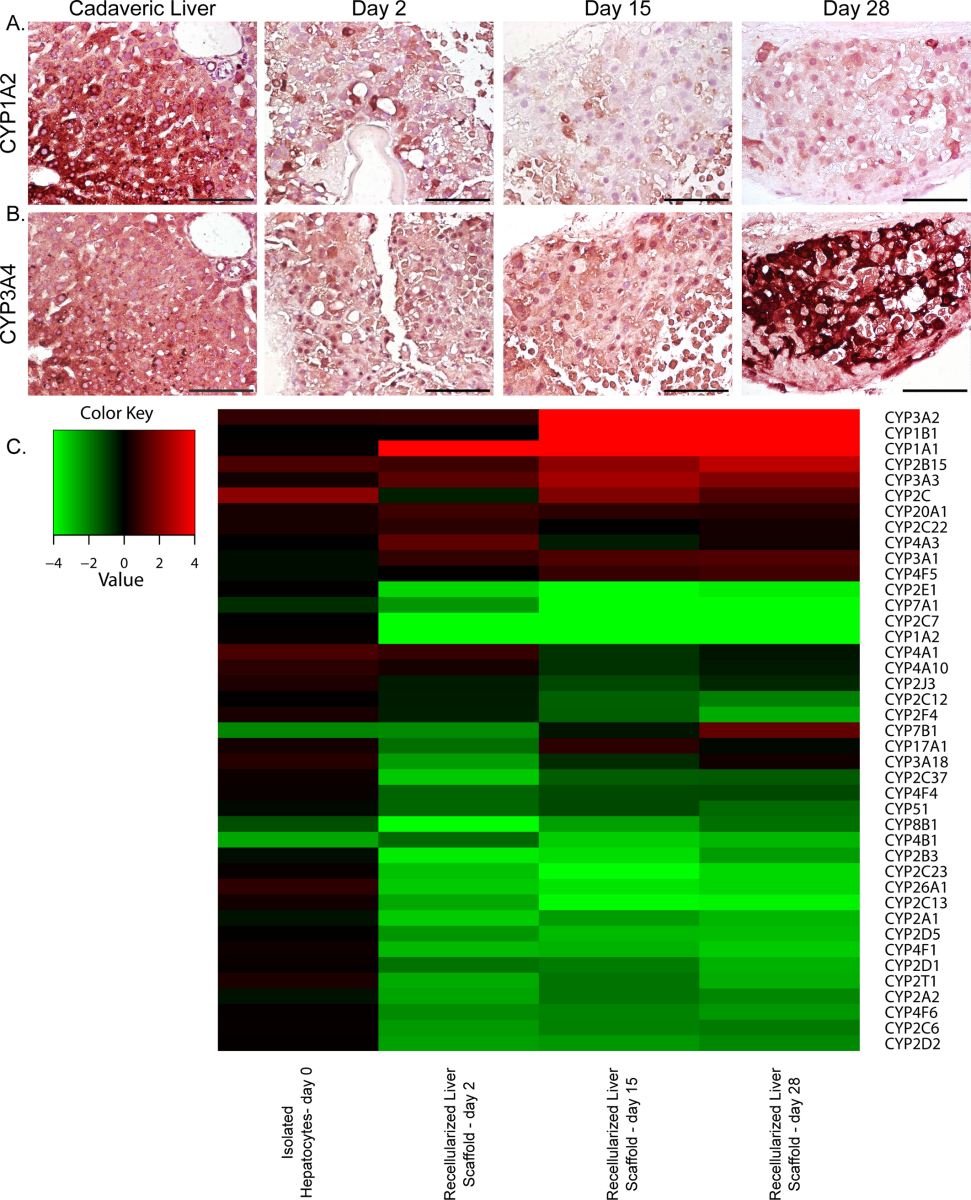
Cytochrome P450 expression in scaffolds recellularized with twenty million rat liver cells. (A,B) Images show CYP1A2 (A) and CYP3A4 (B) expression in cadaveric liver and recellularized liver scaffolds during 28 days in vitro. Scale bars represent 100 microns. (C) Heat map of microarray analysis results showing a 2-fold change in cytochrome P450 expression in freshly isolated hepatocytes and recellularized scaffolds at days 2, 15, and 28 after recellularization.

To further evaluate construct function, we quantified cytochrome P450 expression in liver constructs recellularized with twenty million rat liver cells. Immunohistochemical analysis showed that CYP1A2 expression in the recellularized constructs was decreased, as compared to that in cadaveric liver (Fig 4A). In addition, CYP1A2 expression was no longer restricted to zones near the central vein; instead, it was heterogeneous throughout the recellularized parenchyma. CYP3A4 expression was also no longer zonal in the recellularized scaffolds; however, it increased over time and was more uniform than CYP1A2 expression (Fig 4B). When we performed microarray analysis to examine gene expression levels in the recellularized liver constructs on days 2, 15, and 28 after recellularization, we observed increased expression of genes in the CYP3 family of cytochrome P450 genes, confirming the immunostaining results (Fig 4C). Importantly, these findings show that the liver constructs did not lose their ability to express cytochrome P450 genes, even 28 days after recellularization, indicating that the cells maintained their phenotype.

### Recellularization of the caudate lobes of acellular rat liver scaffolds with human liver cells

**Fig 5:**
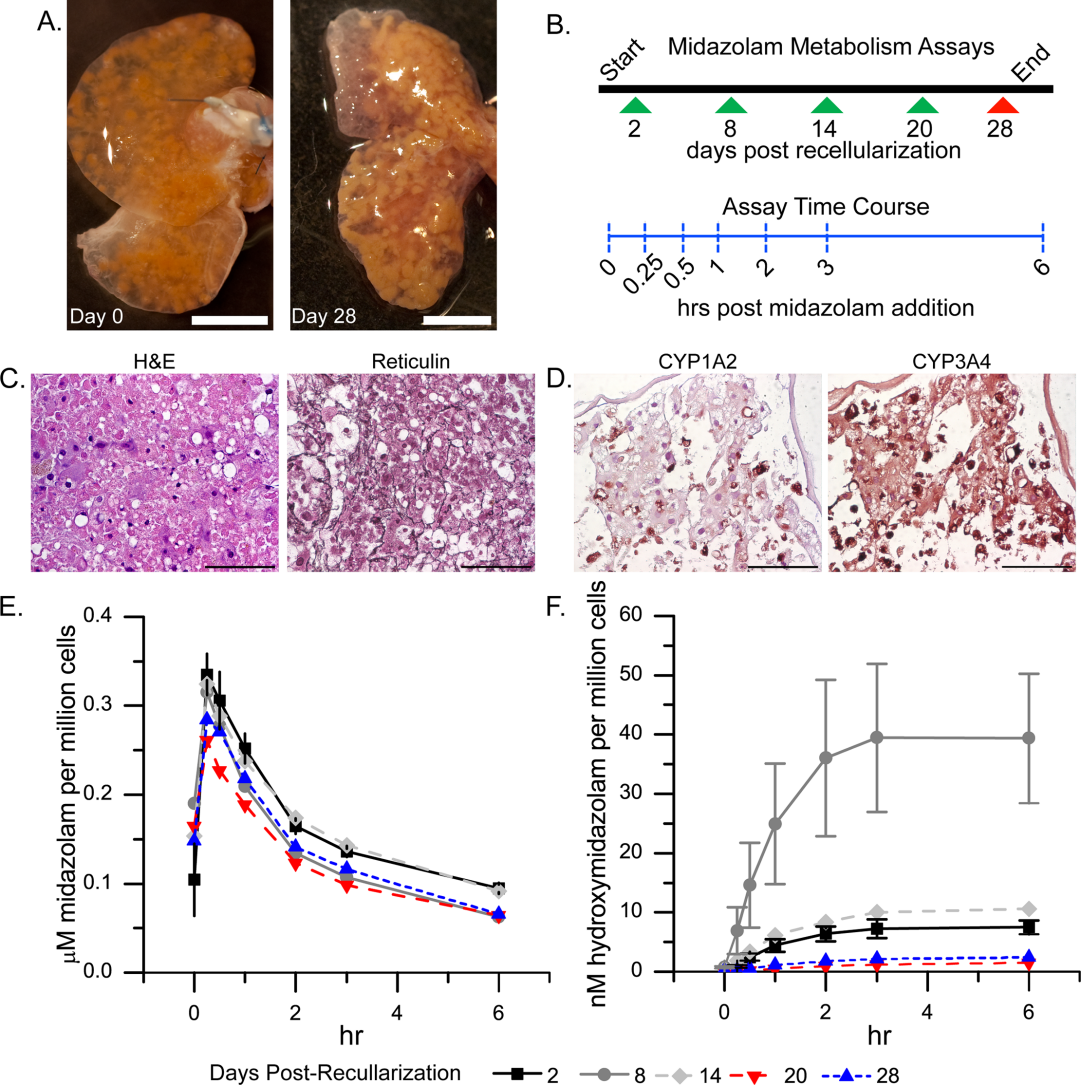
Midazolam drug metabolism over 28 days in rat liver scaffolds recellularized with twenty million human liver cells. (A) Images showing liver scaffolds immediately (day 0) after recellularization and 28 days later. (B) Schematic showing when midazolam metabolism was assayed (arrowheads) and the sampling times during the assay (vertical dashed blue lines). (C) Hematoxylin and eosin (H&E) and reticulin staining 28 days after liver scaffolds were recellularized with human cells. (D) Immunostaining for CYP1A2 and CYP3A4 at day 28 post-recellularization. Scale bars represent 0.5 cm (A) or 100 microns (C and D). (E, F) Metabolism of midazolam (E) and appearance of the metabolite hydroxymidazolam (F). Values shown are the average for 4 constructs. Error bars show the standard error of the mean.

To create liver constructs containing human liver cells, we used a strategy similar to that used for recellularization with rat hepatocytes. Decellularized rat liver caudate lobes were isolated, and twenty million human liver cells were perfused via the portal vein (Fig 5A). Twenty million human liver cells were used to allow direct comparison with rat liver cell recellularization experiments. The human cell constructs were maintained in culture for 28 days. The histologic findings for the liver constructs recellularized with human liver cells for 28 days resembled those for the constructs recellularized with rat liver cells: the cells were retained on the scaffolds and increased reticulin staining was seen surrounding the cells (Fig 5C). Furthermore, like with rat cells, at 28 days after recellularization with human cells, no TUNEL-positive staining was detected, whereas PCNA-positive cells that resembled both hepatocytes and non-parenchymal cell were observed, indicating cell survival, engraftment and proliferation over the month period (S5A Fig). Cell survival was also confirmed by examining G6PDH activity in the cell culture medium. Like the trend observed in AST data for the scaffolds recellularized with rat cells (Fig 3C), the scaffolds recellularized with human liver cells showed an initial increase in G6PDH activity, followed by a decrease and then stabilization in activity (S5B Fig).

### Functional assessment of liver scaffolds recellularized with human liver cells

Cell function was monitored for 28 days after rat liver constructs were recellularized with twenty million human liver cells. These scaffolds retained liver-like function: albumin production peaked at day 12 (S5C Fig); whereas urea concentration initially decreased and then stabilized after 2 weeks (S5D Fig). Furthermore, CYP1A2 and CYP3A4 expression patterns were similar to those observed for the scaffolds recellularized with rat liver cells; at 28 days post-recellularization, CYP1A2 was expressed heterogeneously and sparsely, whereas CYP3A4 was expressed more homogenously (Fig 5D).

To determine CYP activity, capacity of the constructs recellularized with twenty million human liver cells to metabolize midazolam was quantified over a 28-day period. On days 2, 8, 14, 20, and 28 post-recellularization, 10 mL of midazolam-containing medium (non-oxygenated) was circulated through the portal vein of the constructs at a rate of 1 mL/min for 6 hours (Fig 5B and S3C Fig). Perfusate samples were collected from the outlet of the bioreactor at 0, 0.25, 0.5, 1, 2, 3, and 6 hours after midazolam administration and analyzed to determine the concentrations of midazolam and one of its metabolites, hydroxymidazolam. There was an initial lag in the midazolam concentration before it reached a maximum because the bolus of midazolam-containing medium first had to wash out the latent midazolam-free culture medium from the liver lobes. After the maximum concentration was reached, the concentration began to decrease (Fig 5E). The highest concentration of hydroxymidazolam was seen on day 8 after recellularization, whereas the lowest hydroxymidazolam concentrations were seen on days 20 and 28 after recellularization (Fig 5F). There was a statistically significant decrease in the average maximum in midazolam concentration in the cell culture medium on day 20 after recellularization, as compared to the midazolam concentration on the second and fourteenth days of cell culture. In addition, the area under the curve (AUC) for midazolam disappearance was significantly lower on days 8, 20 and 28 after recellularization than on days 2 and 14 (Table 2). However, the average time for eliminating half of the midazolam did not show a statistically significant change during the study (Table 2). These results indicate that the liver constructs maintained their ability to metabolize midazolam throughout the 28-day culture period. The trend of reduced AUC could be attributed to increased hepatocyte number; however, if that were the case, then there should be a continued reduction in the AUC. Instead, there was no significant difference among days 8, 20, and 28 with regards to the AUC. In addition, increased hepatocyte number would not explain the observed dynamics in hydroxymidazolam appearance (Fig 5F).

**Table 2:** Midazolam pharmacokinetics in rat liver scaffolds recellularized with human hepatocytes.

Day	C_max_ (μM)	t_1/2_ (hr)	AUC (μM hr per million cell)
2	6.21 ± 0.40	1.97 ± 0.35	0.96 ± 0.09
8	5.75 ± 0.16	1.35 ± 0.13	0.78 ± 0.05**
14	5.90 ± 0.20	2.03 ± 0.17	0.96 ± 0.08
20	4.76 ± 0.06*	1.61 ± 0.08	0.71 ± 0.03**
28	5.25 ± 0.25	1.78 ± 0.21	0.81 ± 0.04**

Data are shown as mean ± SEM. N = 4 constructs per time point.

*C_max_ on day 20 was statistically significant, as compared to those on days 2 and 14 (*P*<0.05).

**Average AUC on days 8, 20 and 28 were statistically significant, as compared to those on days 2 and 14 (*P*<0.05).

AUC, area under the curve; C_max_, maximum concentration of midazolam; t_1/2_, the half life of midazolam in the media.

### Temporal changes in the phenotype of liver constructs recellularized with rat liver cells

**Fig 6:**
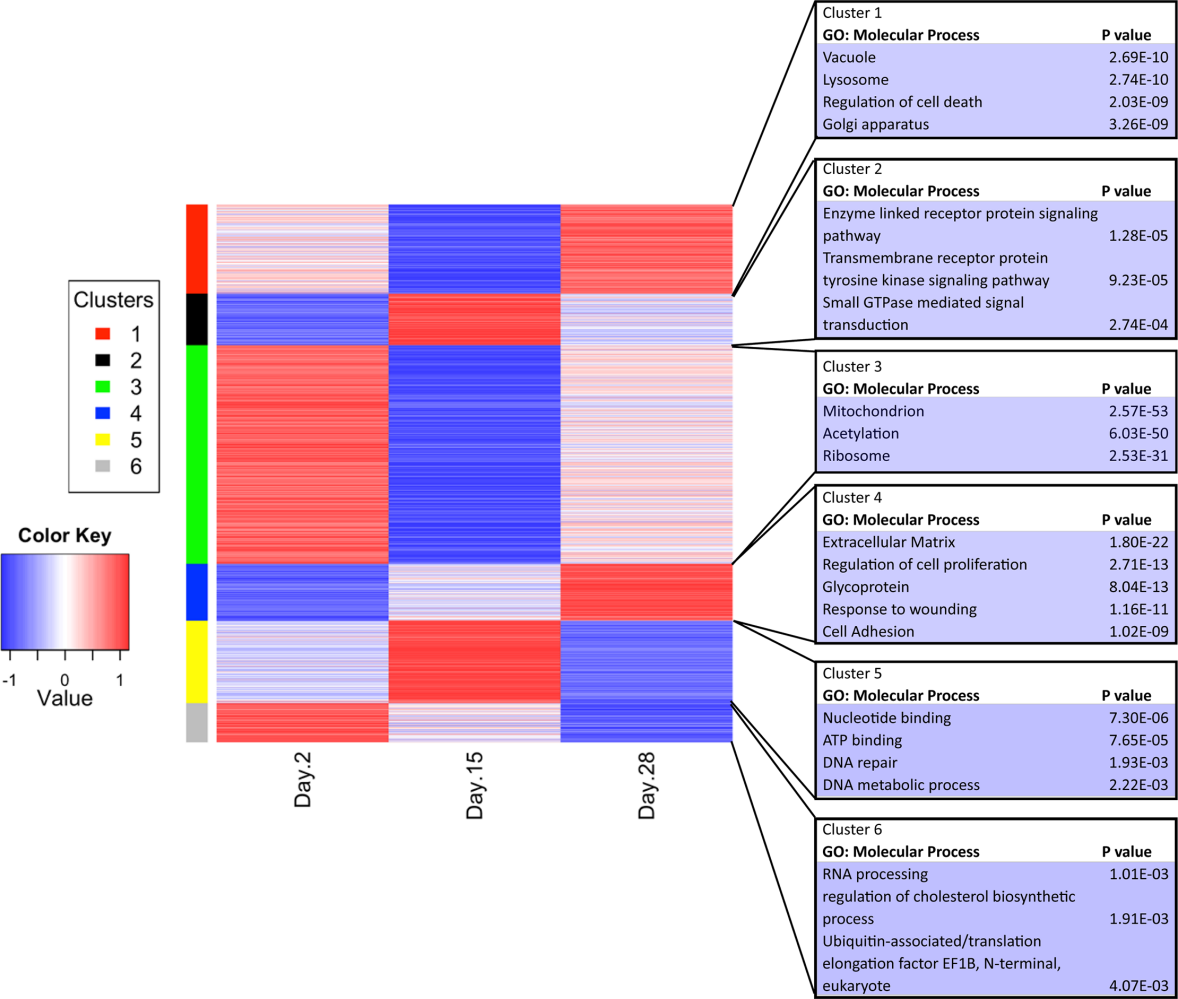
Heat map showing gene expression levels (z-scores) on days 2, 15, and 28 after recellularization with twenty million rat liver cells and clustering of microarray data.

To further assess temporal changes in the phenotype of our liver constructs recellularized with twenty million rat liver cells, we clustered the microarray data from days 2, 15, and 28 post-recellularization. A gene ontology analysis was performed on the expression data from these clusters (Fig 6). Six distinct clusters were generated, with each cluster containing between 600 and 4000 genes. Cluster 1, which represented genes that were significantly decreased on day 15 and increased on day 28, included genes associated with vacuoles, lysosomes, the Golgi apparatus, and regulation of cell death. Cluster 2, which represented genes that were significantly decreased on day 2 and increased on day 15, included genes involved in enzyme-linked receptor protein signaling pathways, transmembrane receptor proteins, tyrosine kinase signaling pathways, and small GTPase-mediated signal transduction pathways. Cluster 3, which represented genes that were significantly increased on day 2 and significantly decreased on day 15, had the most genes, including those associated with mitochondria, acetylation, and ribosomes. This suggests that the cells in the liver constructs showed changes in oxidative phosphorylation, gene transcription, and protein modifications, including epigenetic changes, which may be associated with changes in gene expression. Cluster 4, which represented genes that were significantly decreased on day 2 but significantly increased on day 28, included genes associated with the extracellular matrix, regulation of cell proliferation, glycoproteins, response to wound healing, and cell adhesion. Cluster 5, which represented genes that were increased on day 15 and decreased on day 28, included genes involved in nucleotide binding, ATP binding, DNA repair, and DNA metabolic processes. Lastly, cluster 6, which represented genes that were significantly increased on day 2 and decreased on day 28, included genes involved in RNA processing, regulation of cholesterol biosynthetic processes, and ubiquitin-associated pathways.

Genes important for drug metabolism were present in each cluster (S1 Table). In S2 Table, we list the genes that showed at least a 2-fold increase in expression between day 2 and day 15 (*P*<0.05) and between day 15 and day 28 (*P*<0.05). These genes included extracellular matrix proteins and extracellular matrix remodeling proteins, such as *Igfbp3*, *Mxra8*, *Col3a1*, *Col6a1*, *Bgn*, *Timp2*, *Ogn*, *Snal1*, *Gfpt2*, *Asah3l*, *P4ha1*, *Reck*, *Nrp1*, *Ltbp3*, *Cxcl12*, *Adamsts2*, *Ramp1*, and *Eno2*. Some of these genes have been found to be upregulated in liver fibrosis models. In addition, several of the genes that showed an increase over time have been found to be upregulated in partial hepatectomy liver regeneration models, including *Gja*, *Mgp*, and *Ptprd*. Other genes found to be upregulated included those associated with cell proliferation and regulation of hepatic carcinomas, such as *Emilin1*, *Mmp23b*, *Tmem255b*, *Abi3bp*, *Gpc3*, *Lpar1*, *Efemp2*, *Ogn*, and *Snal*. In contrast, very few genes showed at least a 2-fold significant decrease in expression between day 2 and day 15 and between day 15 and day 28. These gene are listed in the S3 Table.

## Discussion

In our study, we successfully decellularized rat livers using three different procedures: (1) 3.2 L of 1% SDS + 60 mL Triton X-100, (2) 6.4 L of 1% SDS + 60 mL 1% Triton X-100, and (3) 3.2 L 1% SDS + DNase treatment. Of the three perfusion decellularization methods, the third procedure produced scaffolds with the lowest amount of DNA remnants (1.54 ± 0.31%) and the highest amount of GAGs (2073.91 ± 190.2 mg per gram wet sample). This work contributes to the developing body of knowledge about decellularization protocols, which have been used to decellularize livers from a diverse array of species, including mice, rats, ferrets, rabbits, and pigs [13, 20].

In this study, we perfusion-recellularized the caudate lobes of rat livers using adult rat hepatocytes (one million or twenty million cells) or human hepatocytes (twenty million cells). Our recellularized liver constructs maintained a high degree of cellularity for up to 28 days and showed an increase in reticular fibers consisting of type III collagen. We noted an initial period of cell apoptosis, and after that, it decreased with time. By day 28 after recellularization, very few apoptotic cells were observed. In fact, hepatocytes also proliferated within the matrix with the number of PCNA-positive cells peaking at day 15 post-recellularization. We also found that decreasing the number of cells used for recellularization from twenty million to one million led to an increase in cell survival.

A functioning liver will produce albumin and urea. When rat liver cells were used in the recellularization process, albumin and urea production initially declined but stabilized at day 16 post-recellularization, for the remaining two weeks. This decline may reflect a loss in cells as evidenced by TUNEL staining over the first two weeks. Alternatively, the proliferation of cells beginning at about two weeks may have led to a calculation limitation in that activity was normalized to the number of cells actually seeded. Microarray analysis showed no change in albumin gene expression, small changes (less than 2-fold) in enzymes of the urea cycle (ie, ornithine transcarbamylase and argininosuccinate lyase), and small changes (less than 2-fold) in enzymes involved in glucose production (ie, glycogen phosphorylase, UTP glucose-1-phosphate uridylyltransferase 2, 1,4-alpha-glucan branching enzyme, and G6PDH).

When we used human hepatocytes for recellularization, we observed a peak in albumin production at day 12, whereas both urea and G6PDH production had stabilized by that time. In addition, we observed a decrease in AST activity over time, suggesting that the recellularized constructs were stable and that the hepatocytes were not significantly damaged.

Being able to model drug metabolism in vitro is important to the pharmaceutical industry. Eighty-five percent of the drugs that have been removed from the market were removed because of unforeseen drug bioactivation by the liver and subsequent hepatotoxicity [8]. In our study, we assessed liver function by measuring changes in the levels of cytochrome P450 enzymes. Enzymes in the CYP3 family are the most abundant cytochrome P450 enzymes in the liver. CYP3A4, the most abundant isoform within this family, causes 50% of the biotransformations that occur in marketed drugs [8]. In our experiments, the cytochrome P450 enzymes CYP1A2 and CYP3A4 were expressed in livers recellularized with human or rat cells, and this expression was sustained for up to 28 days. In both human cell- and rat cell-recellularized scaffolds, CYP3A4 expression increased over the 28-day period, whereas CYP1A2 expression decreased. The increase in CYP3A4 expression was most likely due to dexamethasone-induced activation of the CYP3A family of cytochrome P450 enzymes [31]. Importantly, the rat and human hepatocytes remained responsive to dexamethasone induction of cytochrome 450 expression even after 28 days in culture. We also evaluated the ability of livers recellularized with human cells to metabolize midazolam. Midazolam, an anesthetic metabolized by liver cytochrome P450 enzymes CYP3A4 and CYP3A5 [32], was added to the cell culture medium perfused through the liver constructs, and the ability of the recellularized livers to metabolize this drug was analyzed. During the experiments, we only measured one of the metabolites formed during midazolam metabolism, hydroxymidazolam. The absolute amount of midazolam that was metabolized by the recellularized livers was similar at days 2, 8, 14, and 28. We used AUC as a surrogate marker of CYP3A4/5 activity and found that by day 28 of culture, the AUC had become smaller, suggesting that CYP3A4/5 activity and the rate of midazolam metabolism had increased. This is possibly because as suggested by our cell proliferation data, the number of hepatocytes increased in this time frame. This cannot be ruled out. However, if this occurred, the cells would likely have de-differentiated to proliferate, and lost their ability to metabolize drugs. Production of the midazolam metabolite hydroxymidazolam was highest on day 8. The microarray data from the liver constructs recellularized with rat liver cells provide 2 possible explanations for this transient increase. First, this could be attributed to an overall increase in CYP3 expression. As suggested in cluster 1 of the microarray data, which showed a pattern of increased gene expression post-recellularization, enriched with enzymes from the CYP3 family. A second possible explanation is that UDP-glucuronosyltransferases (UGT) activity also involved in the metabolism of hydroxymidazolam, was decreased on day 8. In the microarray cluster analysis, UGTs, which are responsible for glucuronic acid conjugation, were enriched in cluster 3, a cluster of genes in which expression was significantly reduced on day 15, as compared to the other time points over the 28-day study period. This transient decrease in UGT expression could account for the increase in hydroxymidazolam expression observed on day 8 because a decreased rate of glucuronic acid addition would slow the metabolism of hydroxymidazolam, thereby increasing the concentration of hydrozymidazolam in the analyte. In the future, it will be important to study the interplay between culture conditions (medium and perfusion rate) and cell phenotype to create a construct with the same cytochrome P450 zonation and expression levels as those found in native liver in vivo.

Although other groups have looked at the expression levels of the cytochrome P450 genes CYP3A4, CYP2C9, and CYP1A1 in recellularized liver constructs [17], we performed microarray analysis on ~5000 genes. In our study, very few genes showed a continual decrease in expression over time, suggesting that the rat cells used to repopulate the liver constructs quickly reached a point of tissue homeostasis and maintained their hepatocyte phenotype rather than de-differentiating. Genes that showed a 2-fold or more decrease in expression over all 3 time points included those associated with type I collagen degradation, thyroid hormone metabolism, and metabolism to produce toxins. In contrast, there were at least 65 genes that showed a statistically significant increase in expression from day 2 to day 15 and from day 15 to day 28. Around 30% of these genes produce extracellular matrix proteins or proteins involved in extracellular matrix remodeling, such as *Igfbp3*, *Mxra8*, *Col3a1*, *Col6a1*, *Bgn*, *Timp2*, *Ogn*, *Snal1*, *Gfpt2*, *Asah3l*, *P4ha1*, *Reck*, *Nrp1*, *Ltbp3*, *Cxcl12*, *Adamsts2*, *Ramp1*, and *Eno2*. Some of these genes have been shown to be important in models of liver fibrosis, such as *Col6a*, *Timp2 Cxcl12 Adamsts2*, *Eno2*, *Snal1*, *Acer2*, and *P4hal*. In addition, several genes that showed an increase in expression over time have been found to be upregulated in partial hepatectomy liver regeneration models; these included *Gja*, *Mgp*, and *Ptprd*. Upregulated genes that have previously been associated with cell proliferation and regulation of hepatic carcinomas included *Emilin1*, *Mmp23b*, *Tmem255b*, *Abi3bp*, *Gpc3*, *Lpar1*, *Efemp2*, *Ogn*, and *Snal*. These data suggest that even though our liver constructs retained the ability to metabolize drugs, they were initiating a regenerative program.

Some (~18%) of the genes that showed a 2-fold increase in expression in the liver constructs from day 2 to day 15 and from day 15 to day 28 have an unknown role in liver function. One example is the extracellular matrix remodeling gene matrix-remodeling associated 8 (*Mxra*), which is known to regulate cell adhesion and matrix remodeling but has never been studied in the context of liver disease. Other genes with no known functions in the liver were also discovered in the microarray analysis, including 2 homeobox genes: HOP homeobox (*Hopx)*, the silencing of which leads to uterine endometrial cancer, and homeobox protein Hox-B2 (*Hoxb2)*, the increased expression of which is associated with pancreatic cancer. A few transmembrane proteins were upregulated, including transmembrane protein 255B *(Tmem255b)*, which inhibits tumor cell proliferation and tumorigenesis in human pancreatic cancer; transmembrane protein 178 (*Tmem178)*, the function of which is unknown; and transmembrane protein with EGF-like and 2 follistatin-like domains 2 *(Tmeff2)*, which is androgen-regulated and has anti-proliferative effects in prostate cancer cells.

Previous studies have demonstrated that decellularized liver matrices can support freshly isolated or immortalized liver cells and that they can also support cells from different species. In addition, it has been shown that cells cultured on decellularized livers can maintain their capacity to metabolize drugs [12, 17–19]. In our experiments, we built upon these findings by demonstrating that the caudate lobes of decellularized rat livers can be recellularized with liver cells from humans or rats. After the cells were seeded onto the acellular scaffolds, they underwent a phase of adjustment, in which some cells (e.g., those that were insufficiently perfused with culture medium or that were mechanically damaged during the cell perfusion process) underwent programmed cell death. Varying cell numbers suggested that a lower initial cell number was superior for cell survival. However, with both numbers, the apoptotic phase was followed by a phase in which some cells showed an upregulation of proliferation markers. The cells then entered a phase of regeneration or repair, in which a significant number of genes associate with liver fibrosis and ECM turnover and synthesis were upregulated. During the first month after recellularization, the cells maintained the capacity to produce albumin and urea and to metabolize a model drug (midazolam).

In conclusion, our results suggest that liver constructs consisting of recellularized rat caudate lobes, recellularized with human hepatocytes acclimated for several days can be used as a model for studying drug metabolism, as well as liver regeneration and fibrosis. In future studies, it will be important to determine whether these recellularized liver constructs, when cultured under physiologic conditions, can establish proper liver zonation and distribution of cytochrome P450 enzymes to better model in vivo drug metabolism. Regardless, our data suggest these in vitro liver constructs can serve as liver surrogates for up to 4 weeks in vitro.

## Supporting information

S1 Fig Gravity perfusion of detergent through the portal vein of the liver removed the cells, generating an acellular scaffold. (A,B) Representative whole rat livers before (A) and after (B) treatment with 3.2 L of 1% SDS and 60 mL of Triton X-100. Scale bars represent 0.5 cm. (C,D) Scanning electron microscopy images of rat livers before (C) and after (D) treatment with 3.2 L of 1% SDS and 60 mL of Triton X-100. Scale bars represent 100 microns.
(TIF)

S2 Fig Components of the rat liver extracellular matrix were retained after detergent decellularization. (A,B) Images show staining for type I collagen (A) and elastin (B) in livers decellularized with 1% SDS and 1% Triton X-100. Scale bars represent 100 microns.
(TIF)

S3 Fig Bioreactors for the recellularized livers were set up within a tissue culture incubator. (A) Image showing a typical bioreactor setup. The numbers correspond to the bioreactor (1), the carbogen humidification flask (2), the medium reservoir (3), and the peristaltic pump used to perfuse the media (4). (B) Diagram detailing how the bioreactor was set up in the incubator for construct maintenance. (C) Diagram showing how the constructs were set up in order to circulate 10 mL of medium during the drug metabolism studies. The arrowhead between the bioreactor and medium reservoir indicates where the medium samples were collected from during the drug metabolism studies.
(TIF)

S4 Fig Reducing the number of rat liver cells perfused into the isolated liver lobes from twenty million to one million resulted in decreased cell death and improved cell health at 2 days post-recellularization. (A-C) Images show hematoxylin and eosin (A), reticulin (B), and TUNEL (C) staining of the recellularized livers. In (C), DAPI-stained cell nuclei are blue and TUNEL-positive cells are red (arrow). Scale bars represent 100 microns.
(TIF)

S5 Fig Acellular rat liver scaffolds were recellularized with human liver cells, cultured for 28 days, and characterized. (A) Images show TUNEL and PCNA staining at 28 days post-recellularization. TUNEL- and PCNA-positive cells are red, and DAPI-stained cell nuclei are blue. Scale bars represent 100 microns. Asterisks (*) indicate PCNA-positive cells. (B-D) Graphs show G6PDH activity (B), albumin production (C), and blood urea nitrogen level (D) in medium samples obtained over a 28-day period from the scaffolds recellularized with human cells. The data points are the average for 4 constructs, and the error bars show the standard error of the mean.
(TIF)

S1 Table Cluster analysis of glucuronosyltransferase and cytochrome P450 expression in constructs recellularized with rat liver cells. (DOCX)

S2 Table Genes that showed at least a 2-fold increase in expression from day 2 to day 15 and then from day 15 to day 28 in constructs recellularized with rat liver cells. (DOCX)

S3 Table Genes that showed at least a 2-fold decrease in expression from day 2 to day 15 and then from day 15 to day 28 in constructs recellularized with rat liver cells. (DOCX)

## References

[1] Yagi H, Tafaleng E, Nagaya M, Hansel MC, Strom SC, Fox IJ, et al Embryonic and induced pluripotent stem cells as a model for liver disease. Crit Rev Biomed Eng. 2009;37(4–5):377–98. Epub 2009/01/01. ; PubMed Central PMCID: PMC3700621. 20528732 10.1615/critrevbiomedeng.v37.i4-5.40 PMC3700621

[2] Tamai M, Yamashita A, Tagawa Y. Mitochondrial development of the in vitro hepatic organogenesis model with simultaneous cardiac mesoderm differentiation from murine induced pluripotent stem cells. J Biosci Bioeng. 2011;112(5):495–500. Epub 2011/08/06. doi: 10.1016/j.jbiosc.2011.07.005 . 21816670 10.1016/j.jbiosc.2011.07.005

[3] Chang R, Emami K, Wu H, Sun W. Biofabrication of a three-dimensional liver micro-organ as an in vitro drug metabolism model. Biofabrication. 2010;2(4):045004 Epub 2010/11/17. doi: 10.1088/1758-5082/2/4/045004 . 21079286 10.1088/1758-5082/2/4/045004

[4] Mueller D, Kramer L, Hoffmann E, Klein S, Noor F. 3D organotypic HepaRG cultures as in vitro model for acute and repeated dose toxicity studies. Toxicol In Vitro. 2014;28(1):104–12. Epub 2013/07/16. doi: 10.1016/j.tiv.2013.06.024 . 23850736 10.1016/j.tiv.2013.06.024

[5] Godoy P, Hewitt NJ, Albrecht U, Andersen ME, Ansari N, Bhattacharya S, et al Recent advances in 2D and 3D in vitro systems using primary hepatocytes, alternative hepatocyte sources and non-parenchymal liver cells and their use in investigating mechanisms of hepatotoxicity, cell signaling and ADME. Archives of toxicology. 2013;87(8):1315–530. Epub 2013/08/27. doi: 10.1007/s00204-013-1078-5 ; PubMed Central PMCID: PMC3753504. 23974980 10.1007/s00204-013-1078-5 PMC3753504

[6] Shackel NA, Gorrell MD, McCaughan GW. Gene array analysis and the liver. Hepatology. 2002;36(6):1313–25. Epub 2002/11/26. doi: 10.1053/jhep.2002.36950 . 12447852 10.1053/jhep.2002.36950

[7] De Bartolo L, Salerno S, Curcio E, Piscioneri A, Rende M, Morelli S, et al Human hepatocyte functions in a crossed hollow fiber membrane bioreactor. Biomaterials. 2009;30(13):2531–43. Epub 2009/02/03. doi: 10.1016/j.biomaterials.2009.01.011 . 19185912 10.1016/j.biomaterials.2009.01.011

[8] Fraczek J, Bolleyn J, Vanhaecke T, Rogiers V, Vinken M. Primary hepatocyte cultures for pharmaco-toxicological studies: at the busy crossroad of various anti-dedifferentiation strategies. Archives of toxicology. 2013;87(4):577–610. Epub 2012/12/18. doi: 10.1007/s00204-012-0983-3 . 23242478 10.1007/s00204-012-0983-3

[9] Lin P, Chan WC, Badylak SF, Bhatia SN. Assessing porcine liver-derived biomatrix for hepatic tissue engineering. Tissue engineering. 2004;10(7–8):1046–53. Epub 2004/09/15. doi: 10.1089/ten.2004.10.1046 . 15363162 10.1089/ten.2004.10.1046

[10] Vasanthan KS, Subramanian A, Krishnan UM, Sethuraman S. Role of biomaterials, therapeutic molecules and cells for hepatic tissue engineering. Biotechnology advances. 2012;30(3):742–52. Epub 2012/01/24. doi: 10.1016/j.biotechadv.2012.01.004 . 22265845 10.1016/j.biotechadv.2012.01.004

[11] Ott HC, Matthiesen TS, Goh SK, Black LD, Kren SM, Netoff TI, et al Perfusion-decellularized matrix: using nature's platform to engineer a bioartificial heart. Nature medicine. 2008;14(2):213–21. doi: 10.1038/nm1684 . 18193059 10.1038/nm1684

[12] Uygun BE, Soto-Gutierrez A, Yagi H, Izamis ML, Guzzardi MA, Shulman C, et al Organ reengineering through development of a transplantable recellularized liver graft using decellularized liver matrix. Nature medicine. 2010;16(7):814–20. Epub 2010/06/15. doi: 10.1038/nm.2170 ; PubMed Central PMCID: PMC2930603. 20543851 10.1038/nm.2170 PMC2930603

[13] Baptista PM, Siddiqui MM, Lozier G, Rodriguez SR, Atala A, Soker S. The use of whole organ decellularization for the generation of a vascularized liver organoid. Hepatology. 2011;53(2):604–17. Epub 2011/01/29. doi: 10.1002/hep.24067 . 21274881 10.1002/hep.24067

[14] De Kock J, Ceelen L, De Spiegelaere W, Casteleyn C, Claes P, Vanhaecke T, et al Simple and quick method for whole-liver decellularization: a novel in vitro three-dimensional bioengineering tool? Archives of toxicology. 2011;85(6):607–12. Epub 2011/04/23. doi: 10.1007/s00204-011-0706-1 . 21512802 10.1007/s00204-011-0706-1

[15] Lang R, Stern MM, Smith L, Liu Y, Bharadwaj S, Liu G, et al Three-dimensional culture of hepatocytes on porcine liver tissue-derived extracellular matrix. Biomaterials. 2011;32(29):7042–52. Epub 2011/07/05. doi: 10.1016/j.biomaterials.2011.06.005 . 21723601 10.1016/j.biomaterials.2011.06.005

[16] Soto-Gutierrez A, Zhang L, Medberry C, Fukumitsu K, Faulk D, Jiang H, et al A whole-organ regenerative medicine approach for liver replacement. Tissue Eng Part C Methods. 2011;17(6):677–86. Epub 2011/03/08. doi: 10.1089/ten.tec.2010.0698 ; PubMed Central PMCID: PMC3103054. 21375407 10.1089/ten.tec.2010.0698 PMC3103054

[17] Zhou P, Lessa N, Estrada DC, Severson EB, Lingala S, Zern MA, et al Decellularized liver matrix as a carrier for the transplantation of human fetal and primary hepatocytes in mice. Liver Transpl. 2011;17(4):418–27. Epub 2011/03/30. doi: 10.1002/lt.22270 ; PubMed Central PMCID: PMC3079538. 21445925 10.1002/lt.22270 PMC3079538

[18] Barakat O, Abbasi S, Rodriguez G, Rios J, Wood RP, Ozaki C, et al Use of decellularized porcine liver for engineering humanized liver organ. The Journal of surgical research. 2012;173(1):e11–25. Epub 2011/11/22. doi: 10.1016/j.jss.2011.09.033 . 22099595 10.1016/j.jss.2011.09.033

[19] Ji R, Zhang N, You N, Li Q, Liu W, Jiang N, et al The differentiation of MSCs into functional hepatocyte-like cells in a liver biomatrix scaffold and their transplantation into liver-fibrotic mice. Biomaterials. 2012;33(35):8995–9008. Epub 2012/09/19. doi: 10.1016/j.biomaterials.2012.08.058 . 22985996 10.1016/j.biomaterials.2012.08.058

[20] Kajbafzadeh AM, Javan-Farazmand N, Monajemzadeh M, Baghayee A. Determining the optimal decellularization and sterilization protocol for preparing a tissue scaffold of a human-sized liver tissue. Tissue Eng Part C Methods. 2013;19(8):642–51. Epub 2012/12/29. doi: 10.1089/ten.TEC.2012.0334 . 23270591 10.1089/ten.TEC.2012.0334

[21] Ren H, Shi X, Tao L, Xiao J, Han B, Zhang Y, et al Evaluation of two decellularization methods in the development of a whole-organ decellularized rat liver scaffold. Liver international: official journal of the International Association for the Study of the Liver. 2013;33(3):448–58. Epub 2013/01/11. doi: 10.1111/liv.12088 . 23301992 10.1111/liv.12088

[22] Song JJ, Guyette JP, Gilpin SE, Gonzalez G, Vacanti JP, Ott HC. Regeneration and experimental orthotopic transplantation of a bioengineered kidney. Nat Med. 2013;19(5):646–51. Epub 2013/04/16. doi: 10.1038/nm.3154 ; PubMed Central PMCID: PMC3650107. 23584091 10.1038/nm.3154 PMC3650107

[23] Uygun BE, Yarmush ML. Engineered liver for transplantation. Current opinion in biotechnology. 2013;24(5):893–9. Epub 2013/06/25. doi: 10.1016/j.copbio.2013.05.008 ; PubMed Central PMCID: PMC3783566. 23791465 10.1016/j.copbio.2013.05.008 PMC3783566

[24] Xia L, Arooz T, Zhang S, Tuo X, Xiao G, Susanto TA, et al Hepatocyte function within a stacked double sandwich culture plate cylindrical bioreactor for bioartificial liver system. Biomaterials. 2012;33(32):7925–32. Epub 2012/08/15. doi: 10.1016/j.biomaterials.2012.06.078 . 22889484 10.1016/j.biomaterials.2012.06.078

[25] Kreamer BL, Staecker JL, Sawada N, Sattler GL, Hsia MT, Pitot HC. Use of a low-speed, iso-density percoll centrifugation method to increase the viability of isolated rat hepatocyte preparations. In Vitro Cell Dev Biol. 1986;22(4):201–11. Epub 1986/04/01. . 2871008 10.1007/BF02623304

[26] Shi W, Oshlack A, Smyth GK. Optimizing the noise versus bias trade-off for Illumina whole genome expression BeadChips. Nucleic acids research. 2010;38(22):e204 Epub 2010/10/12. doi: 10.1093/nar/gkq871 ; PubMed Central PMCID: PMC3001098. 20929874 10.1093/nar/gkq871 PMC3001098

[27] J. van der Laan M, Pollard KS. A new algorithm for hybrid hierarchical clustering with visualization and the bootstrap. Journal of Statistical Planning and Inference. 2003;117(2):275–303.

[28] Huang da W, Sherman BT, Lempicki RA. Bioinformatics enrichment tools: paths toward the comprehensive functional analysis of large gene lists. Nucleic acids research. 2009;37(1):1–13. Epub 2008/11/27. doi: 10.1093/nar/gkn923 ; PubMed Central PMCID: PMC2615629. 19033363 10.1093/nar/gkn923 PMC2615629

[29] Crapo PM, Gilbert TW, Badylak SF. An overview of tissue and whole organ decellularization processes. Biomaterials. 2011;32(12):3233–43. Epub 2011/02/08. doi: 10.1016/j.biomaterials.2011.01.057 ; PubMed Central PMCID: PMC3084613. 21296410 10.1016/j.biomaterials.2011.01.057 PMC3084613

[30] Gilbert TW, Sellaro TL, Badylak SF. Decellularization of tissues and organs. Biomaterials. 2006;27(19):3675–83. Epub 2006/03/08. doi: 10.1016/j.biomaterials.2006.02.014 . 16519932 10.1016/j.biomaterials.2006.02.014

[31] Wright MC, Paine AJ. Induction of the cytochrome P450 3A subfamily in rat liver correlates with the binding of inducers to a microsomal protein. Biochemical and biophysical research communications. 1994;201(2):973–9. Epub 1994/06/15. doi: 10.1006/bbrc.1994.1797 . 8003039 10.1006/bbrc.1994.1797

[32] Gorski JC, Hall SD, Jones DR, VandenBranden M, Wrighton SA. Regioselective biotransformation of midazolam by members of the human cytochrome P450 3A (CYP3A) subfamily. Biochemical pharmacology. 1994;47(9):1643–53. Epub 1994/04/29. . 8185679 10.1016/0006-2952(94)90543-6

