# Supplementary figures and images for: Recellularization of rat liver: An in vitro model for assessing human drug metabolism and liver biology
Source: PLoS One. 2018 Jan 29;13(1):e0191892. doi: 10.1371/journal.pone.0191892 (PMC5788381; doi:10.1371/journal.pone.0191892)

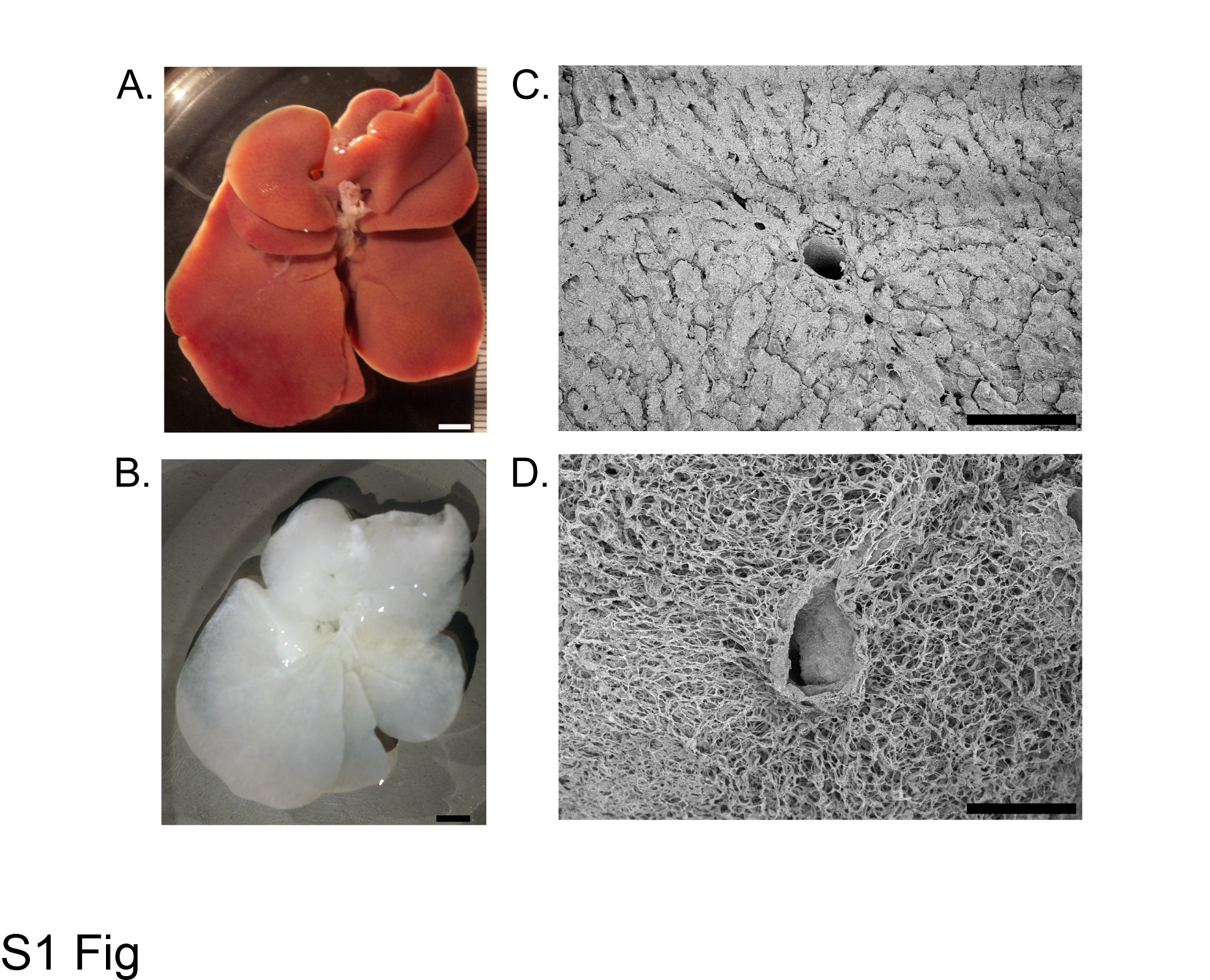

Supplement: S1 Fig — (A,B) Representative whole rat livers before (A) and after (B) treatment with 3.2 L of 1% SDS and 60 mL of Triton X-100. Scale bars represent 0.5 cm. (C,D) Scanning electron microscopy images of rat livers before (C) and after (D) treatment with 3.2 L of 1% SDS and 60 mL of Triton X-100. Scale bars represent 100 microns. (TIF) [file pone.0191892.s001.tif]

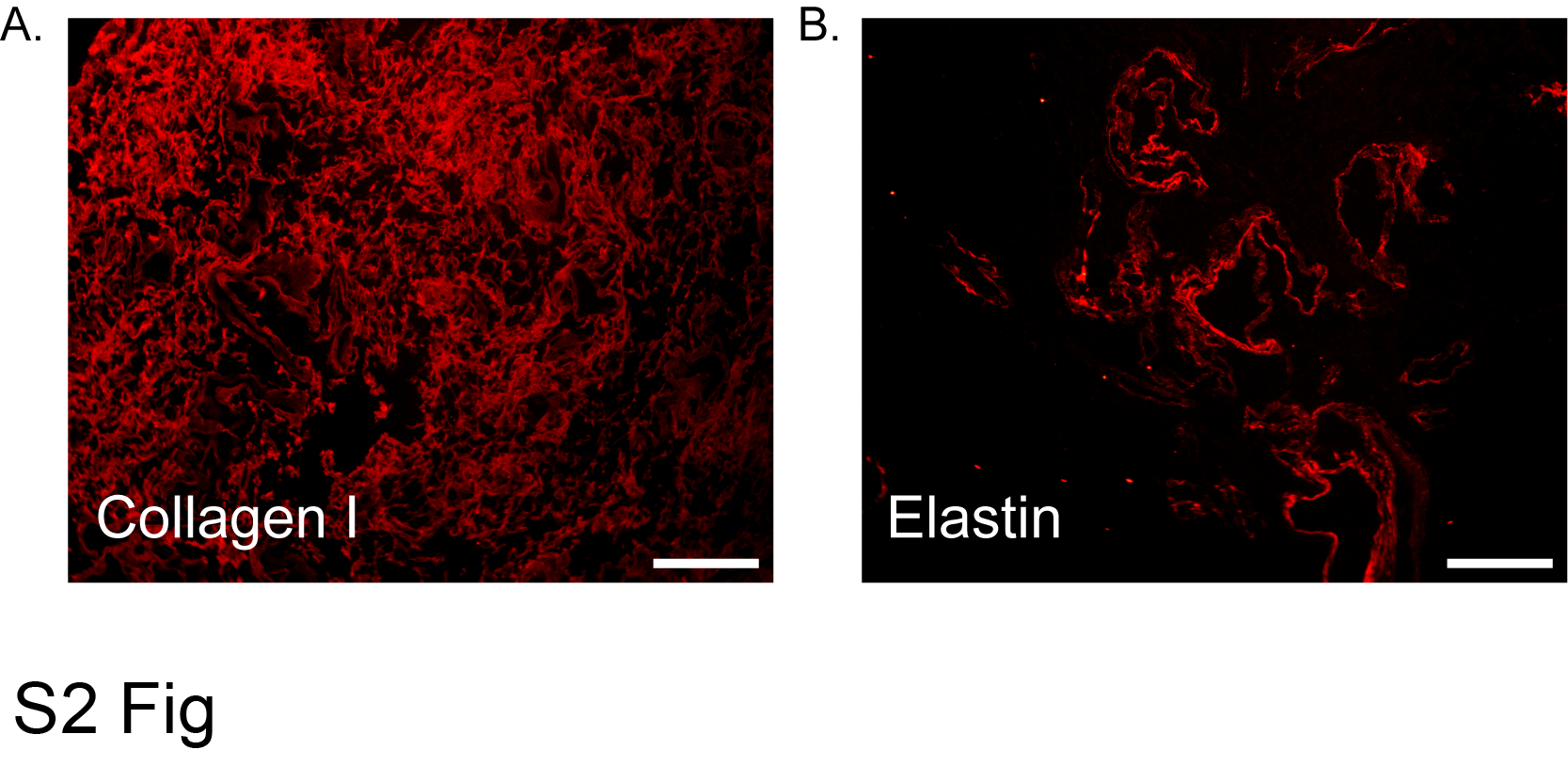

Supplement: S2 Fig — (A,B) Images show staining for type I collagen (A) and elastin (B) in livers decellularized with 1% SDS and 1% Triton X-100. Scale bars represent 100 microns. (TIF) [file pone.0191892.s002.tif]

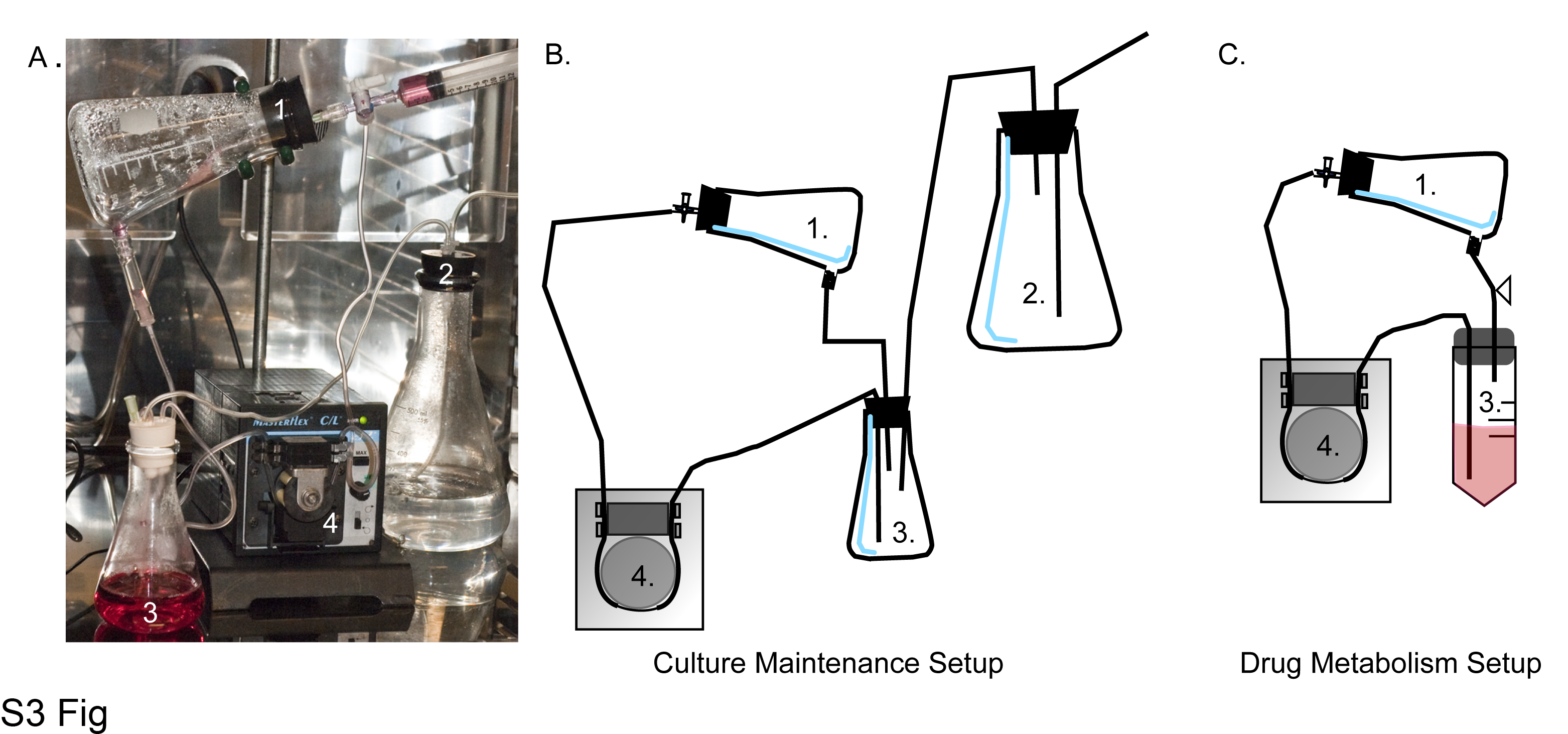

Supplement: S3 Fig — (A) Image showing a typical bioreactor setup. The numbers correspond to the bioreactor (1), the carbogen humidification flask (2), the medium reservoir (3), and the peristaltic pump used to perfuse the media (4). (B) Diagram detailing how the bioreactor was set up in the incubator for construct maintenance. (C) Diagram showing how the constructs were set up in order to circulate 10 mL of medium during the drug metabolism studies. The arrowhead between the bioreactor and medium reservoir indicates where the medium samples were collected from during the drug metabolism studies. (TIF) [file pone.0191892.s003.tif]

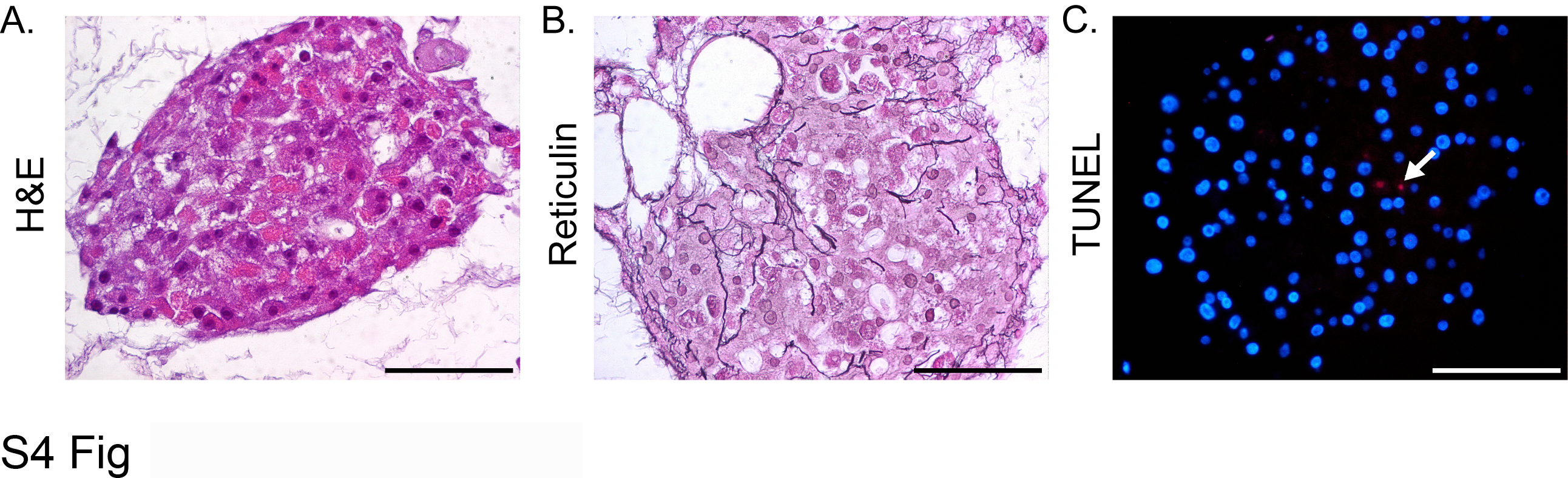

Supplement: S4 Fig — (A-C) Images show hematoxylin and eosin (A), reticulin (B), and TUNEL (C) staining of the recellularized livers. In (C), DAPI-stained cell nuclei are blue and TUNEL-positive cells are red (arrow). Scale bars represent 100 microns. (TIF) [file pone.0191892.s004.tif]

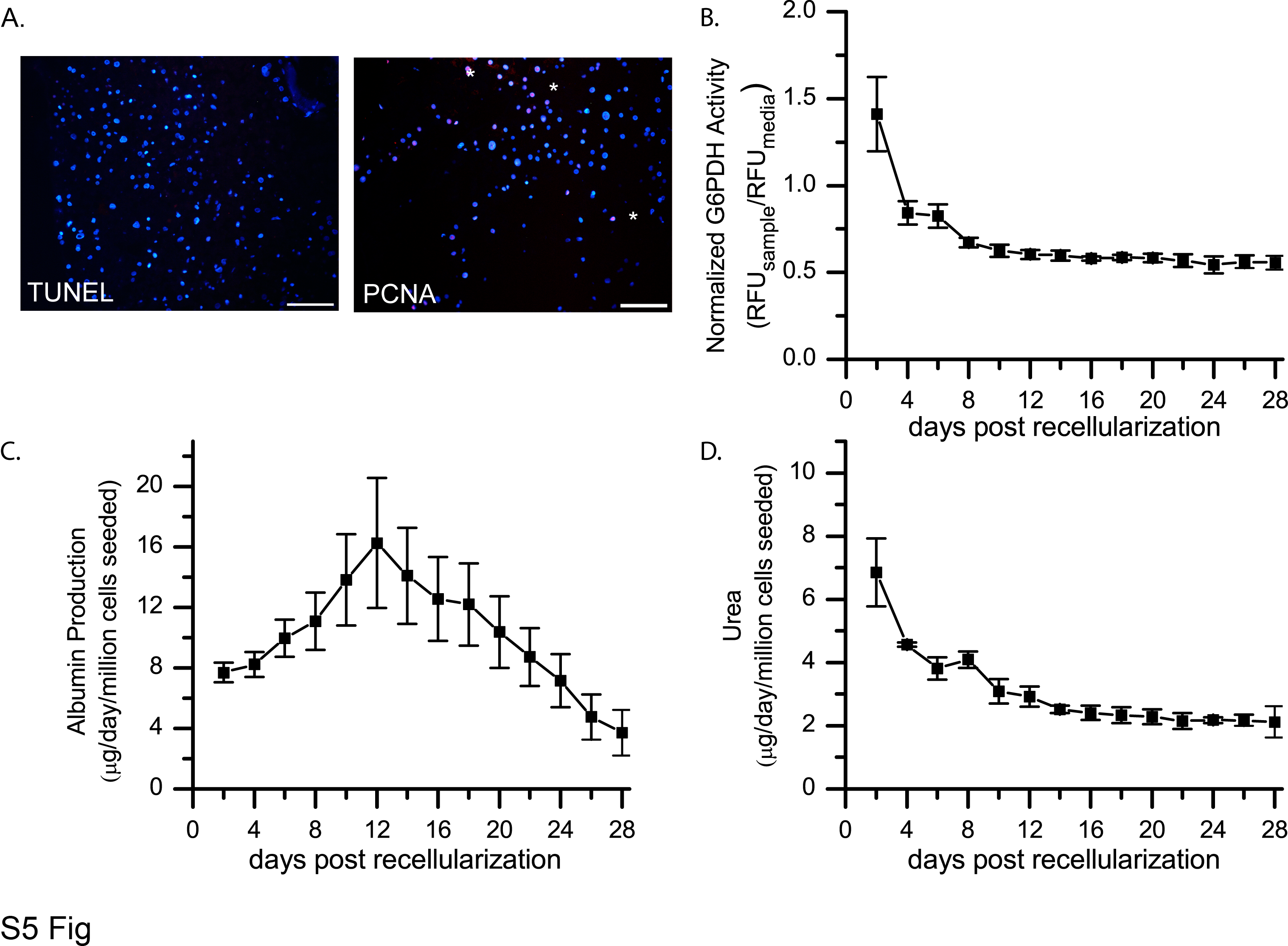

Supplement: S5 Fig — (A) Images show TUNEL and PCNA staining at 28 days post-recellularization. TUNEL- and PCNA-positive cells are red, and DAPI-stained cell nuclei are blue. Scale bars represent 100 microns. Asterisks (*) indicate PCNA-positive cells. (B-D) Graphs show G6PDH activity (B), albumin production (C), and blood urea nitrogen level (D) in medium samples obtained over a 28-day period from the scaffolds recellularized with human cells. The data points are the average for 4 constructs, and the error bars show the standard error of the mean. (TIF) [file pone.0191892.s005.tif]
