# Supplementary material for: Recellularization of rat liver: An in vitro model for assessing human drug metabolism and liver biology
Source: PLoS One. 2018 Jan 29;13(1):e0191892. doi: 10.1371/journal.pone.0191892 (PMC5788381; doi:10.1371/journal.pone.0191892)
Supplement: S1 Table — (DOCX) [file pone.0191892.s006.docx]

**S1 Table. Cluster analysis of glucuronosyltransferase and cytochrome P450 expression in constructs recellularized with rat liver cells**

| **Cluster** | |  | | **Cytochrome P450 enzymes** | |  | | **UDP glucuronosyltransferases** | |
| --- | --- | --- | --- | --- | --- | --- | --- | --- | --- |
| 1 |  | | *CYP2A1, CYP2A2, CYP2B15, CYP2B3,* ***CYP3A1, CYP3A18, CYP3A3****, CYP4F4* | |  | | *UGT2B10* | |  |
|  |  | |  | |  | |  | |  |
| 2 |  | | *CYP2C, CYP4F5* | |  | | *none* | |  |
|  |  | |  | |  | |  | |  |
| 3 |  | | *CYP20A1, CYP27A1, CYP2C12, CYP2C22, CYP2E1, CYP2F4, CYP2J3, CYP2T1,* ***CYP3A13****, CYP4A1, CYP4A10, CYP4A3, CYP4F6, CYP51, CYP7A1* | |  | | *UGT1A1, UGT1A7, UGT2B17, UGT2B36, UGT2B5* | |  |
|  |  | |  | |  | |  | |  |
| 4 |  | | *CYP11A1, CYP17A1, CYP1B1, CYP26B1, CYP2A3A, CYP2C37, CYP2C55, CYP3A2, CYP4A8, CYP7B1, CYP8B1* | |  | | *none* | |  |
|  |  | |  | |  | |  | |  |
| 5 |  | | *CYP2C13, CYP2C23, CYP2D1, CYP2D5, CYP2R1* | |  | | *UGT1A6, UGT1A8* | |  |
|  |  | |  | |  | |  | |  |
| 6 |  | | *CYP2C7, CYP4B1* | |  | | *none* | |  |
