# Supplementary material for: Recellularization of rat liver: An in vitro model for assessing human drug metabolism and liver biology
Source: PLoS One. 2018 Jan 29;13(1):e0191892. doi: 10.1371/journal.pone.0191892 (PMC5788381; doi:10.1371/journal.pone.0191892)
Supplement: S2 Table — (DOCX) [file pone.0191892.s007.docx]

**S2 Table. Genes that showed at least a 2-fold increase in expression from day 2 to day 15 and then from day 15 to day 28 in constructs recellularized with rat liver cells**

| **Gene Symbol** | **Gene Name** | **Description** | **Reference** |
| --- | --- | --- | --- |
| *Igfbp3* | Insulin-like growth factor binding protein 3 | Impedes aggressive growth of pediatric liver cancer; regulates excess collagen expression | [1,2] |
| *Mxra8* | Matrix-remodeling associated 8 | Unknown function in liver; modulates cell adhesion and matrix remodeling | [3] |
| *Col3a1* | Collagen type III alpha | Expression is closely related to chronic liver diseases | [4] |
| *Col6a1* | Collagen type VI, alpha 1 | Highly associated with liver fibrosis | [5] |
| *Bgn* | Biglycan | Core protein of chondroitin/dermatan sulfate proteoglycan; increased expression during liver fibrogenesis | [6] |
| *Timp2* | Tissue inhibitor of metalloproteinase 2 | Increased expression in liver fibrosis; limits matrix degradation | [7] |
| *Gpc3* | Glypican 3 | Potential use as a diagnostic marker of hepatic carcinoma; expressed in normal liver, focal nodular hyperplasia, and liver cirrhosis and increased in hepatic carcinoma | [8] |
| *Ssg1* | Steroid-sensitive Gene-1 | Regulated in liver and small intestine in response to agonists of the pregnane x receptor | [9] |
| *Gja1* | Gap junction protein, alpha 1 | Present in Glisson's capsule; not upregulated after hepatectomy; upregulated in extended cultures of primary hepatocytes and hepatocellular carcinomas, maybe due to increased mRNA stability | [10] |
| *Abcg1* | ATP-binding cassette sub-family G member 1 | Helps regulate cellular cholesterol homeostasis | [11,12] |
| *Cyp3a2* | Cytochrome P450 | Expressed in rat liver; high-dose endotoxin downregulates expression; induction by phenobarbital increases ischemia-reperfusion injury | [13-15] |
| *Cxcl12* | Stromal-derived factor 1 | Induces hepatic stellate cell contraction, possibly promoting portal hypertension; expressed by bile duct epithelial cells in normal liver tissue; upregulated in endothelium in inflammatory foci of HCV- and HBV-associated liver fibrosis | [16,17] |
| *Mgp* | DNA-3 methyladenine glycosylase | DNA repair enzyme; induced during rat liver regeneration after partial hepatectomy | [18] |
| *RGD1565950 (Adamts2)* | A disintegrin and metalloproteinase with thrombospondin motifs 2 | Processes procollagen proteins; reduces the extent of CCl_4_-induced hepatic fibrosis | [19] |
| *Ramp1* | Receptor activity modifying protein 1 | Increased in CCl_4_-induced liver cirrhosis | [20] |
| *Rcn3* | Reticulocalbin 3 | Also called EF-hand calcium binding protein RLP49; member of the CREC family; localizes in the endoplasmic reticulum; contains N-glycosylation sites; may be involved in tumor invasiveness | [21] |
| *Art3* | Ecto-ADP-ribosyltransferase 3 | Expressed in liver; this Art lacks arginine-specific transferase activity | [22] |
| *Lpar1* | Lysophosphatidic acid receptor 1 | Upregulated in organ fibrosis; increased expression in hepatocellular carcinoma | [23,24] |
|  |  |  |  |
| *Efemp2* | Epidermal growth factor-containing fibulin-like extracellular matrix protein 2 | Angiostatic; tumor-suppressor gene; upregulated in hepatocellular carcinoma | [25] |
| *Aebp1* | Adipocyte enhancer-binding protein 1 | Expressed in liver; represses nuclear receptors and cholesterol efflux mediators (ABCA1, ABCG1, and ApoE) in macrophages | [26,27] |
| *Emilin1* | Elastin microfibril interfacer 1 | Belongs to family of glycoproteins of the extracellular matrix; associated with elastic fibers in blood vessels; an adhesive ligand for α4β1 for cell adhesion, migration, and proliferation | [28] |
| *Eno2* | Enolase 2 | Expressed in liver; involved in glycolysis; a decrease in Enolase I is a serum marker of HBV-associated hepatic fibrosis; Enolase 2 is found in neural tissue | [29] |
| *Tacc1* | Transforming acidic coiled-coil-containing protein 1 | Function not clear; may promote cell division | [30] |
| *Cpz* | Carboxypeptidase Z | Zinc-containing exopeptidase that catalyzes the removal of C-terminal amino acids from proteins; secreted and located in the extracellular matrix; may modulate Wnt signaling | [31] |
| *Rhoj* | Ras homolog family member J | Small GTP-binding protein associated with focal adhesions; may regulate angiogenesis and cell motility | [32,33] |
| *Tnfrsf11b* | Tumor necrosis factor receptor superfamily, member 11b | Glycoprotein that is a decoy receptor for the receptor activator of nuclear factor kappa B ligand (RANKL); inhibits inflammation | [34] |
| *Mmp23b* | Matrix metalloproteinase 23b | Promotes liver development and hepatocyte proliferation through the tumor necrosis factor pathway in zebrafish | [35] |
| *Hopx* | HOP homeobox | Unknown function in liver; silencing leads to uterine endometrial cancer | [36] |
| *Prkar2b* | Protein kinase, cAMP-dependent, regulatory, type II, beta | Regulatory subunit of the cAMP-dependent protein kinases involved in cAMP signaling in cells; type II regulatory chains mediate membrane association by binding to anchoring proteins, including the MAP2 kinase | [37] |
| *Ogn* | Osteoglycin | Inhibits gelatinases; decreases metastasis in mouse hepatocarcinoma | [38,39] |
| *Mrap* | Melanocortin 2 receptor accessory protein | G protein-coupled receptor accessory protein; shared function with receptor accessory modifying proteins (RAMPs); traffics melanocortin 2 receptor to plasma membrane to interact with the pituitary hormone ACTH | [40] |
| *Snal1* | Snail homolog 1 | Promotes progression of liver fibrosis; mediates hypoxia-induced epithelial-mesenchymal transition in hepatocellular carcinoma; a TGF beta-regulated transcription factor; aids in epithelial-to-mesenchymal transition during myofibroblastic transformation of rat hepatic cells | [41-45] |
| *Fntb* | Farnesyltransferase subunit beta | Increased expression of farnesyltransferase in primary liver cancer | [46] |
| *Rgs4* | Regulator of G protein signaling 4 | Controls glucose and fatty acid homeostasis | [47] |
|  |  |  |  |
| *Abca9* | ATP-binding cassette sub-family C member 9 | Member of the ABC1 subfamily; expression is suppressed by cholesterol import | [48] |
| *RGD1564008 (Dact1)* | Dapper, antagonist of beta-catenin, homolog 1 | Evolutionarily conserved Dvl-interacting protein that antagonizes Wnt signaling | [49,50] |
| *Prl8a2* | Prolactin family 8, subfamily a, member 2 | Members of the prolactin family are homodimeric tyrosine kinase receptors; metabolic hormone | [51] |
| *Ctdspl* | Carboxy-terminal domain, RNA polymerase II, polypeptide A | Other proteins bind the C-terminal domain of RNA polymerase to induce polymerase activity; involved in the initiation of transcription, the capping of the RNA transcript, and attachment to the spliceosome for RNA splicing | [52] |
| *RGD1566042 (Tmem255b)* | Also FAM70B; Transmembrane protein 255B | Unknown function in liver; inhibits tumor cell proliferation and tumorigenesis in human pancreatic cancer | [53] |
| *Kbtbd9* | Kelch repeat and BTB (POZ) domain-containing 9 | Unknown function in liver; many kelch-repeat proteins are involved in organization of the cytoskeleton via interaction with actin and intermediate filaments | [54] |
| *St3gal2* | ST3 beta-galactoside alpha-2,3-sialyltransferase 2 | Unknown function in liver; a type II membrane protein that catalyzes the transfer of sialic acid from CMP-sialic acid to galactose-containing substrates; a stage-specific embryonic antigen-4 synthase increased in renal carcinogenesis | [55] |
| *Hoxb2* | Homeobox protein Hox-B2 | Unknown function in liver; increased expression is associated with pancreatic cancer | [56] |
| *Tfpi* | Tissue factor pathway inhibitor | Serine proteinase inhibitor; inhibits hepatic ischemia-reperfusion injury; TFPI can increase anticoagulant activity on hepatic sinusoidal walls | [57,58] |
| *Pthr1* | Parathyroid hormone 1 receptor | Member of the secretin family of G protein-coupled receptors; increases in rat liver in response to endotoxin exposure; induces hepatic production of IL-6 and sIL6R | [59,60] |
| *Serpinb1a* | Serpinb1a serine (or cysteine) peptidase inhibitor, clade B, member 1a | Upregulated in Nrf2^-/-^ livers (NFE2-related factor [Nrf2] is a central regulator of detoxification genes); reduces tissue damage caused by proteases during inflammation | [61,62] |
| *Scarf2* | Scavenger receptor class F, member 2 | May account for the high uptake of modified lipoproteins in vivo by liver sinusoidal endothelial cells | [63,64] |
| *Pde10a* | Phosphodiesterase 10A | Unknown function in liver; regulates the intracellular concentration of cyclic nucleotides | [65] |
| *Smad6* | SMAD family member 6 | SMAD6 knockdown mice show liver damage and SMAD6 inhibits non-canonical TGF-beta 1 signaling; suppresses the growth and self-renewal of hepatic progenitor cells; with HDAC3, it inhibits glucocorticoid receptor transcription | [66-68] |
| *RGD1562717 (Abi3bp)* | ABI gene family, member 3 binding protein | Switches mesenchymal stem cells from proliferation to differentiation; exhibits collagen, glycosaminoglycan, and heparin binding | [69] |
| *Itm2c* | Integral membrane protein 2C | Unknown function in liver; integral proteins are located in peroxisomes in livers | [70] |
| *F3* | F3 coagulation factor III (thromboplastin, tissue factor) | Cell surface glycoprotein; expressed in hepatocytes, which may contribute to the early loss of infused cells in liver cell transplantation | [71] |
| *Gfpt2* | Glutamine-fructose-6-phosphate transaminase 2 | Rate-limiting step in nutrient sensing hexosamine pathway; overexpression increased TGF beta in fibrotic complications | [72,73] |
| *Asah3l (Acer2)* | Alkaline Ceramidase 2 | Increased activity in hepatic fibrosis | [74] |
| *St6galnac2* | Alpha-N-acetylgalacrosaminide alpha-2-6-sialyltransferase 2 | A sialytransferase expressed in fetal calf liver | [75] |
| *P4ha1* | Prolyl 4-hydroxylase subunit alpha-1 | Catalyzes the formation of 4-hydroxyproline in collagens; mir-122 decreases P4HA1 and may play a role in limiting liver fibrosis | [76,77] |
| *Reck* | Reversion-inducing-cysteine-rich protein with kazal motifs | Inhibits MMP-2, MMP-9, and MT1-MMP | [78] |
| *Hdc* | Histidine decarboxylase | Found in liver; generates histamine; induced by endotoxin, IL-1, and TNF in liver; inhibition decreases fibrogenesis in patients | [79-82] |
| *Nrp1* | Neuropilin 1 | Promotes cirrhosis of the liver; increases between 24 h and 96 h following hepatectomy | [83,84] |
| *Tmem178* | Transmembrane protein 178 | Unknown function in liver |  |
| *Zcchc12* | Zinc finger, CCHC domain-containing 12 | Interacts with Smads and is a transcriptional coactivator in the bone morphogenetic protein (BMP)-signaling pathway | [85] |
| *Olfml3* | Olfactomedin-like 3 | Proangiogenic cue within the tumor microenvironment | [86] |
| *Ltbp3* | Latent growth factor beta-binding protein 3 | Released by hepatocytes and binds to matrix; thought that binding to matrix is a prerequisite for TGF-beta activation | [87-89] |
| *Bhlhb3*  *(Bhlhe41)* | Class E basic helix-loop-helix protein 41 | Unknown function in liver |  |
| *Abcd2* | ATP-binding cassette, sub-family D (ALD), member 2 (also ATP-binding cassette [ABC] transporter adrenoleukodystrophy-related protein) | Peroxisomal transporter that promotes oxidation of long-chain fatty acids; expression induced upon cholesterol depletion in cultured cells | [90,91] |
| *Tmeff2* | Transmembrane protein with EGF-like and two follistatin-like domains 2 | Unknown function in liver; androgen-regulated and has antiproliferative effects in prostate cancer cells | [92] |

**References**

1. Regel I, Eichenmuller M, Joppien S, Liebl J, Haberle B, Muller-Hocker J, et al. IGFBP3 impedes aggressive growth of pediatric liver cancer and is epigenetically silenced in vascular invasive and metastatic tumors. Mol Cancer. 2012; 11: 9. doi: 10.1186/1476-4598-11-9

2. Flynn RS, Mahavadi S, Murthy KS, Grider JR, Kellum JM, Akbari H, et al. Endogenous IGFBP-3 regulates excess collagen expression in intestinal smooth muscle cells of Crohn's disease strictures. Inflamm Bowel Dis. 2011; 17: 193-201. doi: 10.1002/ibd.21351

3. Walker MG, Volkmuth W. Cell adhesion and matrix remodeling genes identified by co-expression analysis. Gene Function & Disease. 2002; 3: 109-112. doi: 10.1002/gnfd.200290000

4. Lee SK, Yi CH, Kim MH, Cheong JY, Cho SW, Yang SJ, et al. Genetic association between functional haplotype of collagen type III alpha 1 and chronic hepatitis B and cirrhosis in Koreans. Tissue Antigens. 2008; 72: 539-548. doi: 10.1111/j.1399-0039.2008.01144.x

5. Veidal SS, Karsdal MA, Vassiliadis E, Nawrocki A, Larsen MR, Nguyen QH, et al. MMP mediated degradation of type VI collagen is highly associated with liver fibrosis--identification and validation of a novel biochemical marker assay. PLoS One. 2011; 6: e24753. doi: 10.1371/journal.pone.0024753

6. Meyer DH, Krull N, Dreher KL, Gressner AM. Biglycan and decorin gene expression in normal and fibrotic rat liver: Cellular localization and regulatory factors. Hepatology. 1992; 16: 204-216. doi: 10.1002/hep.1840160131

7. Herbst H, Wege T, Milani S, Pellegrini G, Orzechowski HD, Bechstein WO, et al. Tissue inhibitor of metalloproteinase-1 and -2 RNA expression in rat and human liver fibrosis. Am J Pathol. 1997; 150: 1647-1659.

8. Zhu ZW, Friess H, Wang L, Abou-Shady M, Zimmermann A, Lander AD, et al. Enhanced glypican-3 expression differentiates the majority of hepatocellular carcinomas from benign hepatic disorders. Gut. 2001; 48: 558-564.

9. Hartley DP, Dai X, He YD, Carlini EJ, Wang B, Huskey SE, et al. Activators of the rat pregnane X receptor differentially modulate hepatic and intestinal gene expression. Mol Pharmacol. 2004; 65: 1159-1171. doi: 10.1124/mol.65.5.1159

10. Kren BT, Kumar NM, Wang SQ, Gilula NB, Steer CJ. Differential regulation of multiple gap junction transcripts and proteins during rat liver regeneration. J Cell Biol. 1993; 123: 707-718.

11. Wang R, Sheps JA, Ling V. ABC transporters, bile acids, and inflammatory stress in liver cancer. Curr Pharm Biotechnol. 2011; 12: 636-646. doi: 10.2174/138920111795163986

12. Small DM. Role of ABC transporters in secretion of cholesterol from liver into bile. Proc Natl Acad Sci U S A. 2003; 100: 4-6. doi: 10.1073/pnas.0237205100

13. Shaik IH, Mehvar R. Cytochrome P450 induction by phenobarbital exacerbates warm hepatic ischemia-reperfusion injury in rat livers. Free Radic Res. 2010; 44: 441-453. doi: 10.3109/10715761003610729

14. Roe AL, Warren G, Hou G, Howard G, Shedlofsky SI, Blouin RA. The effect of high dose endotoxin on CYP3A2 expression in the rat. Pharm Res. 1998; 15: 1603-1608.

15. Debri K, Boobis AR, Davies DS, Edwards RJ. Distribution and induction of CYP3A1 and CYP3A2 in rat liver and extrahepatic tissues. Biochem Pharmacol. 1995; 50: 2047-2056. doi: 10.1016/0006-2952(95)02107-8

16. Saiman Y, Agarwal R, Hickman DA, Fausther M, El-Shamy A, Dranoff JA, et al. CXCL12 induces hepatic stellate cell contraction through a calcium-independent pathway. Am J Physiol Gastrointest Liver Physiol. 2013; 305: G375-382. doi: 10.1152/ajpgi.00185.2012

17. Wald O, Pappo O, Safadi R, Dagan-Berger M, Beider K, Wald H, et al. Involvement of the CXCL12/CXCR4 pathway in the advanced liver disease that is associated with hepatitis C virus or hepatitis B virus. Eur J Immunol. 2004; 34: 1164-1174. doi: 10.1002/eji.200324441

18. Gombar CT, Katz EJ, Magee PN, Sirover MA. Induction of the DNA repair enzymes uracil DNA glycosylase and 3-methyladenine DNA glycosylase in regenerating rat liver. Carcinogenesis. 1981; 2: 595-599.

19. Kesteloot F, Desmouliere A, Leclercq I, Thiry M, Arrese JE, Prockop DJ, et al. ADAM metallopeptidase with thrombospondin type 1 motif 2 inactivation reduces the extent and stability of carbon tetrachloride-induced hepatic fibrosis in mice. Hepatology. 2007; 46: 1620-1631. doi: 10.1002/hep.21868

20. Hwang IS, Tang F, Leung PP, Li YY, Fan ST, Luk JM. The gene expression of adrenomedullin, calcitonin-receptor-like receptor and receptor activity modifying proteins (RAMPs) in CCl4-induced rat liver cirrhosis. Regul Pept. 2006; 135: 69-77. doi: 10.1016/j.regpep.2006.04.006

21. Honore B, Vorum H. The CREC family, a novel family of multiple EF-hand, low-affinity Ca(2+)-binding proteins localised to the secretory pathway of mammalian cells. FEBS Lett. 2000; 466: 11-18.

22. Glowacki G, Braren R, Firner K, Nissen M, Kuhl M, Reche P, et al. The family of toxin-related ecto-ADP-ribosyltransferases in humans and the mouse. Protein Sci. 2002; 11: 1657-1670. doi: 10.1110/ps.0200602

23. Rancoule C, Pradere JP, Gonzalez J, Klein J, Valet P, Bascands JL, et al. Lysophosphatidic acid-1-receptor targeting agents for fibrosis. Expert Opin Investig Drugs. 2011; 20: 657-667. doi: 10.1517/13543784.2011.566864

24. Sokolov E, Eheim AL, Ahrens WA, Walling TL, Swet JH, McMillan MT, et al. Lysophosphatidic acid receptor expression and function in human hepatocellular carcinoma. J Surg Res. 2013; 180: 104-113. doi: 10.1016/j.jss.2012.10.054

25. Nomoto S, Kanda M, Okamura Y, Nishikawa Y, Qiyong L, Fujii T, et al. Epidermal growth factor-containing fibulin-like extracellular matrix protein 1, EFEMP1, a novel tumor-suppressor gene detected in hepatocellular carcinoma using double combination array analysis. Ann Surg Oncol. 2010; 17: 923-932. doi: 10.1245/s10434-009-0790-0

26. Bogachev O, Majdalawieh A, Pan X, Zhang L, Ro HS. Adipocyte enhancer-binding protein 1 (AEBP1) (a novel macrophage proinflammatory mediator) overexpression promotes and ablation attenuates atherosclerosis in ApoE (-/-) and LDLR (-/-) mice. Mol Med. 2011; 17: 1056-1064. doi: 10.2119/molmed.2011.00141

27. Majdalawieh A, Zhang L, Fuki IV, Rader DJ, Ro HS. Adipocyte enhancer-binding protein 1 is a potential novel atherogenic factor involved in macrophage cholesterol homeostasis and inflammation. Proc Natl Acad Sci U S A. 2006; 103: 2346-2351. doi: 10.1073/pnas.0508139103

28. Colombatti A, Spessotto P, Doliana R, Mongiat M, Bressan GM, Esposito G. The EMILIN/Multimerin family. Front Immunol. 2011; 2: 93. doi: 10.3389/fimmu.2011.00093

29. Zhang B, Wang Z, Deng B, Wu X, Liu J, Feng X. Identification of Enolase 1 and Thrombospondin-1 as serum biomarkers in HBV hepatic fibrosis by proteomics. Proteome Sci. 2013; 11: 30. doi: 10.1186/1477-5956-11-30

30. Lauffart B, Howell SJ, Tasch JE, Cowell JK, Still IH. Interaction of the transforming acidic coiled-coil 1 (TACC1) protein with ch-TOG and GAS41/NuBI1 suggests multiple TACC1-containing protein complexes in human cells. Biochem J. 2002; 363: 195-200.

31. Wang L, Shao YY, Ballock RT. Carboxypeptidase Z (CPZ) links thyroid hormone and Wnt signaling pathways in growth plate chondrocytes. J Bone Miner Res. 2009; 24: 265-273. doi: 10.1359/jbmr.081014

32. Ho H, Soto Hopkin A, Kapadia R, Vasudeva P, Schilling J, Ganesan AK. RhoJ modulates melanoma invasion by altering actin cytoskeletal dynamics. Pigment Cell Melanoma Res. 2013; 26: 218-225. doi: 10.1111/pcmr.12058

33. Leszczynska K, Kaur S, Wilson E, Bicknell R, Heath VL. The role of RhoJ in endothelial cell biology and angiogenesis. Biochem Soc Trans. 2011; 39: 1606-1611. doi: 10.1042/BST20110702

34. Khosla S. Minireview: the OPG/RANKL/RANK system. Endocrinology. 2001; 142: 5050-5055.

35. Qi F, Song J, Yang H, Gao W, Liu NA, Zhang B, et al. Mmp23b promotes liver development and hepatocyte proliferation through the tumor necrosis factor pathway in zebrafish. Hepatology. 2010; 52: 2158-2166. doi: 10.1002/hep.23945

36. Yamaguchi S, Asanoma K, Takao T, Kato K, Wake N. Homeobox gene HOPX is epigenetically silenced in human uterine endometrial cancer and suppresses estrogen-stimulated proliferation of cancer cells by inhibiting serum response factor. Int J Cancer. 2009; 124: 2577-2588. doi: 10.1002/ijc.24217

37. Keryer G, Luo Z, Cavadore JC, Erlichman J, Bornens M. Phosphorylation of the regulatory subunit of type II beta cAMP-dependent protein kinase by cyclin B/p34cdc2 kinase impairs its binding to microtubule-associated protein 2. Proc Natl Acad Sci U S A. 1993; 90: 5418-5422.

38. Cui X, Song B, Hou L, Wei Z, Tang J. High expression of osteoglycin decreases the metastatic capability of mouse hepatocarcinoma Hca-F cells to lymph nodes. Acta Biochim Biophys Sin (Shanghai). 2008; 40: 349-355.

39. Cui XN, Tang JW, Song B, Wang B, Chen SY, Hou L. High expression of osteoglycin decreases gelatinase activity of murine hepatocarcinoma Hca-F cells. World J Gastroenterol. 2009; 15: 6117-6122.

40. Webb TR, Clark AJ. Minireview: the melanocortin 2 receptor accessory proteins. Mol Endocrinol. 2010; 24: 475-484. doi: 10.1210/me.2009-0283

41. Choi SS, Omenetti A, Witek RP, Moylan CA, Syn WK, Jung Y, et al. Hedgehog pathway activation and epithelial-to-mesenchymal transitions during myofibroblastic transformation of rat hepatic cells in culture and cirrhosis. Am J Physiol Gastrointest Liver Physiol. 2009; 297: G1093-1106. doi: 10.1152/ajpgi.00292.2009

42. Li X, Deng W, Nail CD, Bailey SK, Kraus MH, Ruppert JM, et al. Snail induction is an early response to Gli1 that determines the efficiency of epithelial transformation. Oncogene. 2006; 25: 609-621. doi: 10.1038/sj.onc.1209077

43. Rowe RG, Lin Y, Shimizu-Hirota R, Hanada S, Neilson EG, Greenson JK, et al. Hepatocyte-derived Snail1 propagates liver fibrosis progression. Mol Cell Biol. 2011; 31: 2392-2403. doi: 10.1128/MCB.01218-10

44. Zavadil J, Bottinger EP. TGF-beta and epithelial-to-mesenchymal transitions. Oncogene. 2005; 24: 5764-5774. doi: 10.1038/sj.onc.1208927

45. Zhang L, Huang G, Li X, Zhang Y, Jiang Y, Shen J, et al. Hypoxia induces epithelial-mesenchymal transition via activation of SNAI1 by hypoxia-inducible factor -1alpha in hepatocellular carcinoma. BMC Cancer. 2013; 13: 108. doi: 10.1186/1471-2407-13-108

46. Sui GD, Zhang GY, Niu ZJ, Hu SY. Expression of farnesyltransferase in primary liver cancer. Chin Med J (Engl). 2012; 125: 2427-2431.

47. Iankova I, Chavey C, Clape C, Colomer C, Guerineau NC, Grillet N, et al. Regulator of G protein signaling-4 controls fatty acid and glucose homeostasis. Endocrinology. 2008; 149: 5706-5712. doi: 10.1210/en.2008-0717

48. Piehler A, Kaminski WE, Wenzel JJ, Langmann T, Schmitz G. Molecular structure of a novel cholesterol-responsive A subclass ABC transporter, ABCA9. Biochem Biophys Res Commun. 2002; 295: 408-416.

49. Waxman JS, Hocking AM, Stoick CL, Moon RT. Zebrafish Dapper1 and Dapper2 play distinct roles in Wnt-mediated developmental processes. Development. 2004; 131: 5909-5921. doi: 10.1242/dev.01520

50. Zhang L, Gao X, Wen J, Ning Y, Chen YG. Dapper 1 antagonizes Wnt signaling by promoting dishevelled degradation. J Biol Chem. 2006; 281: 8607-8612. doi: 10.1074/jbc.M600274200

51. Ben-Jonathan N, Hugo ER, Brandebourg TD, LaPensee CR. Focus on prolactin as a metabolic hormone. Trends Endocrinol Metab. 2006; 17: 110-116. doi: 10.1016/j.tem.2006.02.005

52. Brickey WJ, Greenleaf AL. Functional studies of the carboxy-terminal repeat domain of Drosophila RNA polymerase II in vivo. Genetics. 1995; 140: 599-613.

53. Wang H, Li M, Fisher W, Brunicardi F, Yao Q, Chen C. A Novel Gene FAM70B Inhibits Tumor Cell Proliferation And Tumorigenesis In Human Pancreatic Cancer. Journal of Surgical Research. 2011; 165: 336.

54. Stogios PJ, Prive GG. The BACK domain in BTB-kelch proteins. Trends Biochem Sci. 2004; 29: 634-637. doi: 10.1016/j.tibs.2004.10.003

55. Saito S, Aoki H, Ito A, Ueno S, Wada T, Mitsuzuka K, et al. Human alpha2,3-sialyltransferase (ST3Gal II) is a stage-specific embryonic antigen-4 synthase. J Biol Chem. 2003; 278: 26474-26479. doi: 10.1074/jbc.M213223200

56. Segara D, Biankin AV, Kench JG, Langusch CC, Dawson AC, Skalicky DA, et al. Expression of HOXB2, a retinoic acid signaling target in pancreatic cancer and pancreatic intraepithelial neoplasia. Clin Cancer Res. 2005; 11: 3587-3596. doi: 10.1158/1078-0432.CCR-04-1813

57. Yamanobe F, Mochida S, Ohno A, Ishikawa K, Fujiwara K. Recombinant human tissue factor pathway inhibitor as a possible anticoagulant targeting hepatic sinusoidal walls. Thromb Res. 1997; 85: 493-501.

58. Yoshimura N, Kobayashi Y, Nakamura K, Yamagishi H, Oka T. The effect of tissue factor pathway inhibitor on hepatic ischemic reperfusion injury of the rat. Transplantation. 1999; 67: 45-53.

59. Funk JL, Moser AH, Grunfeld C, Feingold KR. Parathyroid hormone-related protein is induced in the adult liver during endotoxemia and stimulates the hepatic acute phase response. Endocrinology. 1997; 138: 2665-2673.

60. Mitnick MA, Grey A, Masiukiewicz U, Bartkiewicz M, Rios-Velez L, Friedman S, et al. Parathyroid hormone induces hepatic production of bioactive interleukin-6 and its soluble receptor. Am J Physiol Endocrinol Metab. 2001; 280: E405-412.

61. Chartoumpekis DV, Ziros PG, Zaravinos A, Iskrenova RP, Psyrogiannis AI, Kyriazopoulou VE, et al. Hepatic gene expression profiling in Nrf2 knockout mice after long-term high-fat diet-induced obesity. Oxid Med Cell Longev. 2013; 2013: 340731. doi: 10.1155/2013/340731

62. Gong D, Farley K, White M, Hartshorn KL, Benarafa C, Remold-O'Donnell E. Critical role of serpinB1 in regulating inflammatory responses in pulmonary influenza infection. J Infect Dis. 2011; 204: 592-600. doi: 10.1093/infdis/jir352

63. Terpstra V, van Amersfoort ES, van Velzen AG, Kuiper J, van Berkel TJ. Hepatic and extrahepatic scavenger receptors: function in relation to disease. Arterioscler Thromb Vasc Biol. 2000; 20: 1860-1872.

64. Van Berkel TJ, De Rijke YB, Kruijt JK. Different fate in vivo of oxidatively modified low density lipoprotein and acetylated low density lipoprotein in rats. Recognition by various scavenger receptors on Kupffer and endothelial liver cells. J Biol Chem. 1991; 266: 2282-2289.

65. Lau JK, Cheng YK. An update view on the substrate recognition mechanism of phosphodiesterases: a computational study of PDE10 and PDE4 bound with cyclic nucleotides. Biopolymers. 2012; 97: 910-922. doi: 10.1002/bip.22104

66. Ding Z-y, Liang H-f, Jin G-n, Chen W-x, Wang W, Datta PK, et al. Smad6 suppresses the growth and self-renewal of hepatic progenitor cells. Journal of Cellular Physiology. 2013: n/a-n/a. doi: 10.1002/jcp.24488

67. Ichijo T, Voutetakis A, Cotrim AP, Bhattachryya N, Fujii M, Chrousos GP, et al. The Smad6-histone deacetylase 3 complex silences the transcriptional activity of the glucocorticoid receptor: potential clinical implications. J Biol Chem. 2005; 280: 42067-42077. doi: 10.1074/jbc.M509338200

68. Jung SM, Lee JH, Park J, Oh YS, Lee SK, Park JS, et al. Smad6 inhibits non-canonical TGF-beta1 signalling by recruiting the deubiquitinase A20 to TRAF6. Nat Commun. 2013; 4: 2562. doi: 10.1038/ncomms3562

69. Hodgkinson CP, Naidoo V, Patti KG, Gomez JA, Schmeckpeper J, Zhang Z, et al. Abi3bp is a multifunctional autocrine/paracrine factor that regulates mesenchymal stem cell biology. Stem Cells. 2013; 31: 1669-1682. doi: 10.1002/stem.1416

70. Crane DI, Chen N, Masters C. Changes to the integral membrane protein composition of mouse liver peroxisomes in response to the peroxisome proliferators clofibrate, Wy-14,643 and di(2-ethyl-hexyl)phthalate. Mol Cell Biochem. 1988; 81: 29-36.

71. Stephenne X, Vosters O, Najimi M, Beuneu C, Dung KN, Wijns W, et al. Tissue factor-dependent procoagulant activity of isolated human hepatocytes: relevance to liver cell transplantation. Liver Transpl. 2007; 13: 599-606. doi: 10.1002/lt.21128

72. Weigert C, Brodbeck K, Lehmann R, Haring HU, Schleicher ED. Overexpression of glutamine:fructose-6-phosphate-amidotransferase induces transforming growth factor-beta1 synthesis in NIH-3T3 fibroblasts. FEBS Lett. 2001; 488: 95-99.

73. Weigert C, Klopfer K, Kausch C, Brodbeck K, Stumvoll M, Haring HU, et al. Palmitate-induced activation of the hexosamine pathway in human myotubes: increased expression of glutamine:fructose-6-phosphate aminotransferase. Diabetes. 2003; 52: 650-656.

74. Weber SN, Hall RA, Lammert F, Teufel A. Independent genetic associations of hepatic fibrosis and hepatocellular carcinoma in both murine reference and human populations. Z Gastroenterol. 2009; 47: P104. doi: 10.1055/s-0029-1241355

75. Samyn-Petit B, Krzewinski-Recchi MA, Steelant WF, Delannoy P, Harduin-Lepers A. Molecular cloning and functional expression of human ST6GalNAc II. Molecular expression in various human cultured cells. Biochim Biophys Acta. 2000; 1474: 201-211.

76. Li J, Ghazwani M, Zhang Y, Lu J, Fan J, Gandhi CR, et al. miR-122 regulates collagen production via targeting hepatic stellate cells and suppressing P4HA1 expression. J Hepatol. 2013; 58: 522-528. doi: 10.1016/j.jhep.2012.11.011

77. Pihlajaniemi T, Myllyla R, Kivirikko KI. Prolyl 4-hydroxylase and its role in collagen synthesis. J Hepatol. 1991; 13 Suppl 3: S2-7.

78. Dong Q, Yu D, Yang CM, Jiang B, Zhang H. Expression of the reversion-inducing cysteine-rich protein with Kazal motifs and matrix metalloproteinase-14 in neuroblastoma and the role in tumour metastasis. Int J Exp Pathol. 2010; 91: 368-373. doi: 10.1111/j.1365-2613.2010.00724.x

79. Endo Y, Kikuchi T, Nakamura M. Ornithine and histidine decarboxylase activities in mice sensitized to endotoxin, interleukin-1 or tumour necrosis factor by D-galactosamine. Br J Pharmacol. 1992; 107: 888-894.

80. Hocker M, Zhang Z, Koh TJ, Wang TC. The regulation of histidine decarboxylase gene expression. Yale J Biol Med. 1996; 69: 21-33.

81. Ishii K, Suzuki O, Maruyama K, Nagata H, Kiryu Y, Tsuchiya M. Therapeutic effect of histidine decarboxylase inhibitor on chronic active hepatitis. Gastroenterol Jpn. 1978; 13: 105-110.

82. Tran VT, Snyder SH. Histidine decarboxylase. Purification from fetal rat liver, immunologic properties, and histochemical localization in brain and stomach. J Biol Chem. 1981; 256: 680-686.

83. Cao S, Yaqoob U, Das A, Shergill U, Jagavelu K, Huebert RC, et al. Neuropilin-1 promotes cirrhosis of the rodent and human liver by enhancing PDGF/TGF-beta signaling in hepatic stellate cells. J Clin Invest. 2010; 120: 2379-2394. doi: 10.1172/JCI41203

84. Fu L, Kitamura T, Iwabuchi K, Ichinose S, Yanagida M, Ogawa H, et al. Interplay of neuropilin-1 and semaphorin 3A after partial hepatectomy in rats. World J Gastroenterol. 2012; 18: 5034-5041. doi: 10.3748/wjg.v18.i36.5034

85. Li H, Liu Q, Hu X, Feng D, Xiang S, He Z, et al. Human ZCCHC12 activates AP-1 and CREB signaling as a transcriptional co-activator. Acta Biochim Biophys Sin (Shanghai). 2009; 41: 535-544.

86. Miljkovic-Licina M, Hammel P, Garrido-Urbani S, Lee BP, Meguenani M, Chaabane C, et al. Targeting olfactomedin-like 3 inhibits tumor growth by impairing angiogenesis and pericyte coverage. Mol Cancer Ther. 2012; 11: 2588-2599. doi: 10.1158/1535-7163.MCT-12-0245

87. Bissell DM, Roulot D, George J. Transforming growth factor beta and the liver. Hepatology. 2001; 34: 859-867. doi: 10.1053/jhep.2001.28457

88. Hyytiainen M, Penttinen C, Keski-Oja J. Latent TGF-beta binding proteins: extracellular matrix association and roles in TGF-beta activation. Crit Rev Clin Lab Sci. 2004; 41: 233-264. doi: 10.1080/10408360490460933

89. Rifkin DB. Latent transforming growth factor-beta (TGF-beta) binding proteins: orchestrators of TGF-beta availability. J Biol Chem. 2005; 280: 7409-7412. doi: 10.1074/jbc.R400029200

90. Liu J, Liang S, Liu X, Brown JA, Newman KE, Sunkara M, et al. The absence of ABCD2 sensitizes mice to disruptions in lipid metabolism by dietary erucic acid. J Lipid Res. 2012; 53: 1071-1079. doi: 10.1194/jlr.M022160

91. Weinhofer I, Forss-Petter S, Zigman M, Berger J. Cholesterol regulates ABCD2 expression: implications for the therapy of X-linked adrenoleukodystrophy. Hum Mol Genet. 2002; 11: 2701-2708.

92. Gery S, Sawyers CL, Agus DB, Said JW, Koeffler HP. TMEFF2 is an androgen-regulated gene exhibiting antiproliferative effects in prostate cancer cells. Oncogene. 2002; 21: 4739-4746. doi: 10.1038/sj.onc.1205142
