# Supplementary material for: Recellularization of rat liver: An in vitro model for assessing human drug metabolism and liver biology
Source: PLoS One. 2018 Jan 29;13(1):e0191892. doi: 10.1371/journal.pone.0191892 (PMC5788381; doi:10.1371/journal.pone.0191892)
Supplement: S3 Table — (DOCX) [file pone.0191892.s008.docx]

**S3 Table. Genes that showed at least a 2-fold decrease in expression from day 2 to day 15 and then from day 15 to day 28 in constructs recellularized with rat liver cells**

| **Gene symbol** | **Gene name** | **Description** | **Reference** |
| --- | --- | --- | --- |
| *Prss32* | Protease, serine, 32 | Capacity to degrade type I collagen | [1] |
| *Mettl7b* | Methyltransferase-like 7B | Protein associated with lipid droplets in fatty liver | [2] |
| *Dio1* | Iodothronine deiodinase type I | Converts the prohormone thyroxine (T4) to the active thyroid hormone 3,3',5-triiodothyronine (T3); highly expressed in rat liver | [3] |
| *RGD1561090*  *(Ptprd or PTPdelta)* | Protein tyrosine phosphatase, receptor type, D (delta) | Involved in rat liver regeneration; only increased at day 7 post-partial hepatectomy | [4] |
| *Cesl1* | Carboxylesterase-like 1 | Expressed in liver and responsible for the metabolism of drug and environmental esters | [5] |

**References**

1. LeBleu VS, Teng Y, O'Connell JT, Charytan D, Muller GA, Muller CA, et al. Identification of human epididymis protein-4 as a fibroblast-derived mediator of fibrosis. Nat Med. 2013; 19: 227-231. doi: 10.1038/nm.2989

2. Turro S, Ingelmo-Torres M, Estanyol JM, Tebar F, Fernandez MA, Albor CV, et al. Identification and characterization of associated with lipid droplet protein 1: A novel membrane-associated protein that resides on hepatic lipid droplets. Traffic. 2006; 7: 1254-1269.

3. Schoenmakers CH, Pigmans IG, Visser TJ. Species differences in liver type I iodothyronine deiodinase. Biochim Biophys Acta. 1992; 1121: 160-166.

4. Kitamura T, Nakamura K, Mizuno Y, Kikuchi K. Gene expressions of protein tyrosine phosphatases in regenerating rat liver and rat ascites hepatoma cells. Jpn J Cancer Res. 1995; 86: 811-818.

5. Brzezinski MR, Spink BJ, Dean RA, Berkman CE, Cashman JR, Bosron WF. Human liver carboxylesterase hCE-1: binding specificity for cocaine, heroin, and their metabolites and analogs. Drug Metab Dispos. 1997; 25: 1089-1096.
